# Supplementary material for: The COVID-19 pandemic and health-related quality of life across 13 high- and low-middle-income countries: A cross-sectional analysis
Source: PLoS Med. 2023 Apr 11;20(4):e1004146. doi: 10.1371/journal.pmed.1004146 (PMC10089360; doi:10.1371/journal.pmed.1004146)
Supplement: S2 Table — (DOCX) [file pmed.1004146.s002.docx]

**S2 Table. Socio-demographic and clinical characteristics, overall and by country**

|  | **Pooled** | **Australia** | **Brazil** | **Canada** | **Chile** | **China** | **Colombia** | **France** |
| --- | --- | --- | --- | --- | --- | --- | --- | --- |
| **No. sampled - N** | 15,480 | 1,358 | 1,421 | 1,148 | 1,120 | 1,291 | 1,231 | 1,142 |
| **Gender - % (95% CI)** |  |  |  |  |  |  |  |  |
| Male | 51.49 (50.40-52.58) | 46.01 (43.18-48.87) | 49.23 (45.94-52.53) | 53.75 (50.85-56.61) | 41.92 (34.04-50.23) | 61.44 (56.26-66.38) | 43.75 (39.82-47.77) | 47.41 (44.33-50.50) |
| Female & Other | 48.51 (47.42-49.60) | 53.99 (51.13-56.82) | 50.77 (47.47-54.06) | 46.25 (43.39-49.15) | 58.08 (49.77-65.96) | 38.56 (33.62-43.74) | 56.25 (52.23-60.18) | 52.59 (49.50-55.67) |
| Missing | 0.00 (0.00-0.00) | 0.00 (0.00-0.00) | 0.00 (0.00-0.00) | 0.00 (0.00-0.00) | 0.00 (0.00-0.00) | 0.00 (0.00-0.00) | 0.00 (0.00-0.00) | 0.00 (0.00-0.00) |
| **Age (years) - Mean (SD)** | 44.14 (16.49) | 46.01 (17.47) | 42.19 (15.34) | 46.43 (17.20) | 43.04 (15.95) | 50.06 (15.34) | 42.78 (15.93) | 48.28 (16.71) |
| **Education - % (95% CI)** |  |  |  |  |  |  |  |  |
| Primary or less | 23.56 (22.27-24.90) | 25.67 (22.96-28.59) | 43.00 (39.56-46.51) | 5.05 (3.93-6.48) | 23.67 (14.12-36.92) | 74.70 (71.35-77.77) | 45.27 (41.12-49.48) | 16.69 (14.16-19.57) |
| Secondary | 41.27 (40.26-42.29) | 46.35 (43.51-49.22) | 35.00 (32.12-38.00) | 47.74 (44.86-50.63) | 43.66 (36.66-50.92) | 13.74 (11.57-16.24) | 31.57 (28.61-34.69) | 31.51 (29.01-34.13) |
| University | 33.22 (32.35-34.10) | 26.60 (24.52-28.78) | 14.71 (13.40-16.13) | 46.17 (43.30-49.06) | 29.57 (24.85-34.76) | 9.90 (8.70-11.25) | 21.97 (19.76-24.35) | 50.08 (46.97-53.20) |
| Missing | 1.95 (1.75-2.18) | 1.37 (0.88-2.14) | 7.29 (6.06-8.73) | 1.05 (0.59-1.83) | 3.10 (2.17-4.40) | 1.67 (1.09-2.54) | 1.19 (0.72-1.98) | 1.71 (1.11-2.64) |
| **Employment - % (95% CI)** |  |  |  |  |  |  |  |  |
| Employed | 47.08 (46.01-48.16) | 45.62 (42.81-48.46) | 46.27 (43.02-49.56) | 53.05 (50.15-55.92) | 29.62 (24.02-35.91) | 43.46 (38.35-48.73) | 53.41 (49.43-57.34) | 0.00 (0.00-0.00) |
| Unemployed | 9.71 (9.17-10.27) | 8.29 (6.80-10.07) | 17.82 (15.36-20.57) | 7.06 (5.71-8.69) | 7.41 (5.72-9.55) | 1.93 (0.93-3.96) | 12.87 (10.52-15.65) | 0.00 (0.00-0.00) |
| Pension/capital income | 11.87 (10.97-12.84) |  | 13.07 (10.93-15.54) | 21.95 (19.65-24.44) | 10.15 (4.12-22.89) | 34.15 (29.14-39.54) | 5.12 (3.19-8.12) | 0.00 (0.00-0.00) |
| Other | 19.60 (18.63-20.61) | 40.77 (37.96-43.64) | 17.20 (14.81-19.89) | 16.38 (14.35-18.63) | 47.57 (39.78-55.48) | 20.46 (15.67-26.26) | 23.99 (20.81-27.49) | 0.00 (0.00-0.00) |
| Missing | 11.74 (11.13-12.39) | 5.32 (4.11-6.87) | 5.64 (4.36-7.27) | 1.57 (0.99-2.47) | 5.24 (2.30-11.49) | 0.00 (0.00-0.00) | 4.61 (3.16-6.67) | 100.00 (100.00-100.00) |
| **Loss of income due to COVID-19 - % (95% CI)** |  |  |  |  |  |  |  |  |
| Yes | 43.07 (41.97-44.18) | 27.15 (24.76-29.68) | 50.70 (47.41-53.99) | 33.01 (30.35-35.79) | 65.64 (58.60-72.05) | 26.02 (21.39-31.26) | 69.91 (66.21-73.37) | 18.52 (16.21-21.07) |
| No | 52.28 (51.18-53.38) | 68.49 (65.85-71.02) | 42.43 (39.22-45.70) | 63.68 (60.85-66.41) | 28.12 (22.88-34.05) | 72.67 (67.42-77.35) | 25.24 (22.01-28.78) | 77.21 (74.49-79.72) |
| Don't know | 2.36 (1.97-2.83) | 1.81 (1.22-2.69) | 3.02 (2.04-4.43) | 1.48 (0.92-2.37) | 3.42 (0.85-12.76) | 1.10 (0.51-2.37) | 1.70 (0.99-2.90) | 2.18 (1.45-3.27) |
| Missing | 2.29 (2.04-2.57) | 2.55 (1.77-3.64) | 3.85 (2.78-5.31) | 1.83 (1.20-2.79) | 2.82 (1.92-4.12) | 0.21 (0.09-0.51) | 3.15 (2.01-4.90) | 2.09 (1.37-3.18) |
| **Believed to have had COVID-19 - % (95% CI)** |  |  |  |  |  |  |  |  |
| Yes | 15.98 (15.31-16.68) | 9.93 (8.51-11.55) | 23.52 (20.84-26.44) | 8.10 (6.66-9.83) | 9.51 (7.45-12.07) | 5.31 (3.50-7.96) | 21.20 (18.23-24.51) | 12.83 (10.87-15.08) |
| No | 69.42 (68.49-70.34) | 84.44 (82.44-86.26) | 50.03 (46.74-53.32) | 79.53 (77.10-81.77) | 71.96 (65.69-77.49) | 93.87 (91.09-95.83) | 57.34 (53.46-61.13) | 79.60 (76.96-82.00) |
| Don't know | 10.75 (10.12-11.42) | 0.00 (0.00-0.00) | 26.45 (23.67-29.43) | 12.37 (10.59-14.40) | 18.52 (13.81-24.40) | 0.82 (0.30-2.25) | 21.46 (18.60-24.62) | 0.00 (0.00-0.00) |
| Missing | 3.84 (3.53-4.18) | 5.63 (4.49-7.03) | 0.00 (0.00-0.00) | 0.00 (0.00-0.00) | 0.00 (0.00-0.00) | 0.00 (0.00-0.00) | 0.00 (0.00-0.00) | 7.58 (6.10-9.38) |
| **Tested positive for COVID-19 - % (95% CI)** |  |  |  |  |  |  |  |  |
| Yes | 10.53 (9.89-11.22) | 8.54 (7.20-10.11) | 14.42 (12.26-16.89) | 3.75 (2.79-5.01) | 8.13 (4.71-13.68) | 5.92 (3.32-10.34) | 11.98 (9.35-15.23) | 9.20 (7.58-11.12) |
| No | 87.65 (86.94-88.33) | 89.13 (87.37-90.67) | 83.44 (80.83-85.76) | 94.95 (93.52-96.07) | 91.22 (85.76-94.72) | 93.11 (88.74-95.86) | 86.27 (82.97-89.02) | 88.93 (86.85-90.73) |
| Don't know | 0.87 (0.71-1.06) | 0.00 (0.00-0.00) | 2.13 (1.34-3.39) | 1.31 (0.79-2.16) | 0.65 (0.33-1.27) | 0.97 (0.40-2.36) | 1.75 (1.04-2.92) | 0.00 (0.00-0.00) |
| Missing | 0.95 (0.79-1.13) | 2.33 (1.62-3.33) | 0.00 (0.00-0.00) | 0.00 (0.00-0.00) | 0.00 (0.00-0.00) | 0.00 (0.00-0.00) | 0.00 (0.00-0.00) | 1.87 (1.17-2.98) |
| **Relative infected with COVID-19 - % (95% CI)** |  |  |  |  |  |  |  |  |
| Yes | 30.07 (29.01-31.14) | 11.00 (9.48-12.72) | 47.92 (44.65-51.21) | 15.42 (13.44-17.62) | 41.51 (33.36-50.15) | 6.76 (4.70-9.62) | 46.83 (42.87-50.83) | 27.22 (24.52-30.09) |
| No | 64.92 (63.83-65.99) | 85.62 (83.69-87.36) | 44.86 (41.60-48.17) | 79.88 (77.46-82.10) | 54.93 (46.58-63.00) | 91.32 (88.29-93.62) | 46.56 (42.67-50.48) | 69.00 (66.02-71.82) |
| Don't know | 3.37 (3.02-3.76) | 0.00 (0.00-0.00) | 7.22 (5.65-9.18) | 4.70 (3.62-6.09) | 3.57 (1.64-7.58) | 1.92 (1.09-3.36) | 6.61 (5.07-8.59) | 0.00 (0.00-0.00) |
| Missing | 1.65 (1.44-1.88) | 3.39 (2.56-4.47) | 0.00 (0.00-0.00) | 0.00 (0.00-0.00) | 0.00 (0.00-0.00) | 0.00 (0.00-0.00) | 0.00 (0.00-0.00) | 3.79 (2.72-5.24) |
| **Friend/colleague infected with COVID-19 - % (95% CI)** |  |  |  |  |  |  |  |  |
| Yes | 42.37 (41.33-43.42) | 13.18 (11.52-15.04) | 74.14 (71.05-77.02) | 21.86 (19.57-24.35) | 47.77 (40.07-55.58) | 5.11 (3.32-7.79) | 53.16 (49.19-57.09) | 41.08 (38.05-44.18) |
| No | 51.75 (50.67-52.83) | 82.88 (80.81-84.78) | 19.61 (17.01-22.49) | 71.78 (69.10-74.31) | 46.03 (37.95-54.33) | 92.95 (90.03-95.07) | 38.14 (34.34-42.10) | 54.10 (50.98-57.19) |
| Don't know | 3.93 (3.59-4.32) | 0.00 (0.00-0.00) | 6.25 (4.80-8.10) | 6.36 (5.08-7.93) | 6.19 (4.54-8.40) | 1.94 (1.03-3.63) | 8.70 (6.75-11.15) | 0.00 (0.00-0.00) |
| Missing | 1.95 (1.72-2.20) | 3.94 (3.03-5.10) | 0.00 (0.00-0.00) | 0.00 (0.00-0.00) | 0.00 (0.00-0.00) | 0.00 (0.00-0.00) | 0.00 (0.00-0.00) | 4.81 (3.63-6.36) |
| **Know of someone dead from COVID-19 - % (95% CI)** |  |  |  |  |  |  |  |  |
| Yes | 37.02 (35.94-38.10) | 12.66 (11.01-14.51) | 60.48 (57.21-63.66) | 15.85 (13.85-18.08) | 49.23 (41.31-57.20) | 6.39 (4.32-9.35) | 57.76 (53.84-61.59) | 18.31 (16.04-20.83) |
| No | 59.77 (58.68-60.85) | 84.63 (82.62-86.44) | 36.07 (32.96-39.31) | 81.88 (79.55-84.00) | 48.96 (41.13-56.83) | 92.56 (89.59-94.73) | 39.68 (35.90-43.59) | 78.30 (75.63-80.76) |
| Don't know | 1.58 (1.37-1.81) | 0.00 (0.00-0.00) | 3.44 (2.42-4.88) | 2.26 (1.55-3.31) | 1.81 (1.09-2.99) | 1.06 (0.61-1.81) | 2.56 (1.73-3.77) | 0.00 (0.00-0.00) |
| Missing | 1.64 (1.43-1.87) | 2.71 (1.98-3.71) | 0.00 (0.00-0.00) | 0.00 (0.00-0.00) | 0.00 (0.00-0.00) | 0.00 (0.00-0.00) | 0.00 (0.00-0.00) | 3.38 (2.40-4.76) |
| **Comorbidities - % (95% CI)** |  |  |  |  |  |  |  |  |
| Diabetes | 11.73 (10.84-12.67) | 13.98 (12.10-16.09) | 11.41 (9.32-13.90) | 11.60 (9.84-13.63) | 15.78 (8.55-27.31) | 5.03 (3.12-8.01) | 8.18 (5.63-11.74) | 9.22 (7.58-11.18) |
| Hypertension | 18.94 (17.97-19.95) | 23.72 (21.31-26.31) | 18.54 (15.93-21.46) | 20.85 (18.56-23.35) | 23.27 (15.75-32.98) | 18.21 (14.46-22.68) | 15.96 (12.76-19.78) | 13.97 (11.91-16.31) |
| Heart disease | 4.50 (4.11-4.92) | 5.48 (4.28-6.99) | 2.51 (1.54-4.07) | 3.45 (2.52-4.70) | 3.42 (2.25-5.18) | 2.78 (1.39-5.49) | 3.10 (1.82-5.22) | 2.62 (1.78-3.83) |
| Asthma | 8.44 (7.90-9.00) | 15.41 (13.33-17.75) | 5.32 (4.09-6.90) | 10.61 (8.92-12.57) | 6.33 (4.66-8.53) | 4.66 (2.64-8.11) | 5.99 (4.03-8.81) | 5.84 (4.51-7.54) |
| Allergies^+^ | 18.17 (17.39-18.98) | 18.68 (16.54-21.02) | 15.04 (12.85-17.53) | 24.12 (21.68-26.73) | 20.93 (16.73-25.87) | 12.38 (8.85-17.07) | 19.94 (16.75-23.57) | 7.88 (6.33-9.76) |
| Kidney disease | 2.16 (1.77-2.63) | 1.65 (1.05-2.57) | 1.85 (1.01-3.36) | 1.45 (0.89-2.35) | 3.87 (1.25-11.39) | 2.57 (1.22-5.34) | 2.00 (1.19-3.34) | 0.22 (0.07-0.72) |
| Other condition | 9.01 (8.37-9.69) | 16.02 (13.89-18.40) | 3.39 (2.38-4.80) | 10.24 (8.59-12.18) | 17.02 (11.78-23.95) | 1.61 (0.76-3.40) | 4.78 (3.45-6.59) | 6.40 (4.99-8.19) |
| No comorbidity | 49.55 (48.45-50.66) | 43.85 (41.01-46.73) | 41.94 (38.67-45.29) | 48.41 (45.47-51.37) | 38.69 (31.91-45.94) | 62.07 (56.45-67.39) | 58.99 (54.87-63.00) | 53.85 (50.67-56.99) |

N=sample size; %=weighted percentage; Mean=weighted mean; SD=weighted standard deviation**.**

**S2 Table (Continued). Socio-demographic and clinical characteristics, overall and by country**

|  | **Pooled** | **India** | **Italy** | **Spain** | **UK** | **US** | **Uganda** |
| --- | --- | --- | --- | --- | --- | --- | --- |
| **No. sampled - N** | 15,480 | 1,190 | 1,080 | 1,152 | 1,163 | 1,146 | 1,038 |
| **Gender - % (95% CI)** |  |  |  |  |  |  |  |
| Male | 51.49 (50.40-52.58) | 60.50 (57.70-63.25) | 46.45 (43.31-49.62) | 48.61 (45.73-51.50) | 50.15 (47.14-53.17) | 48.81 (45.48-52.15) | 73.41 (70.64-76.01) |
| Female & Other | 48.51 (47.42-49.60) | 39.50 (36.75-42.30) | 53.55 (50.38-56.69) | 51.39 (48.50-54.27) | 49.85 (46.83-52.86) | 51.19 (47.85-54.52) | 26.59 (23.99-29.36) |
| Missing | 0.00 (0.00-0.00) | 0.00 (0.00-0.00) | 0.00 (0.00-0.00) | 0.00 (0.00-0.00) | 0.00 (0.00-0.00) | 0.00 (0.00-0.00) | 0.00 (0.00-0.00) |
| **Age (years) - Mean (SD)** | 44.14 (16.49) | 33.24 (11.59) | 49.05 (15.65) | 47.51 (14.50) | 47.85 (16.75) | 46.93 (17.01) | 29.11 (7.02) |
| **Education - % (95% CI)** |  |  |  |  |  |  |  |
| Primary or less | 23.56 (22.27-24.90) | 27.56 (25.10-30.17) | 3.71 (2.54-5.41) | 9.55 (7.98-11.39) | 7.59 (6.43-8.94) | 3.91 (2.84-5.36) | 3.85 (2.84-5.21) |
| Secondary | 41.27 (40.26-42.29) | 22.61 (20.32-25.07) | 76.03 (73.49-78.40) | 39.93 (37.14-42.79) | 59.45 (56.54-62.29) | 63.44 (60.45-66.33) | 31.89 (29.12-34.79) |
| University | 33.22 (32.35-34.10) | 49.83 (46.99-52.67) | 18.43 (16.46-20.57) | 49.39 (46.51-52.28) | 31.36 (28.73-34.12) | 31.62 (28.96-34.40) | 62.91 (59.93-65.80) |
| Missing | 1.95 (1.75-2.18) | 0.00 (0.00-0.00) | 1.83 (1.22-2.74) | 1.13 (0.66-1.93) | 1.60 (1.02-2.50) | 1.03 (0.59-1.81) | 1.35 (0.80-2.26) |
| **Employment - % (95% CI)** |  |  |  |  |  |  |  |
| Employed | 47.08 (46.01-48.16) | 72.77 (70.17-75.23) | 54.19 (51.00-57.34) | 59.98 (57.12-62.78) | 56.39 (53.39-59.35) | 52.99 (49.62-56.34) | 43.06 (40.08-46.10) |
| Unemployed | 9.71 (9.17-10.27) | 5.38 (4.23-6.81) | 11.14 (9.37-13.21) | 13.63 (11.77-15.73) | 6.61 (5.27-8.27) | 7.95 (6.10-10.31) | 26.97 (24.36-29.76) |
| Pension/capital income | 11.87 (10.97-12.84) | 2.02 (1.36-2.99) | 9.59 (7.71-11.86) | 16.93 (14.87-19.20) | 21.93 (19.64-24.41) | 17.64 (15.23-20.34) | 0.19 (0.05-0.77) |
| Other | 19.60 (18.63-20.61) | 18.57 (16.46-20.88) | 18.47 (16.03-21.20) | 8.07 (6.63-9.79) | 13.86 (11.80-16.21) | 19.50 (16.83-22.47) | 5.49 (4.26-7.05) |
| Missing | 11.74 (11.13-12.39) | 1.26 (0.76-2.08) | 6.60 (5.11-8.50) | 1.39 (0.85-2.26) | 1.20 (0.71-2.04) | 1.92 (1.20-3.07) | 24.28 (21.76-26.98) |
| **Loss of income due to COVID-19 - % (95% CI)** |  |  |  |  |  |  |  |
| Yes | 43.07 (41.97-44.18) | 60.25 (57.44-63.00) | 32.11 (29.31-35.04) | 36.37 (33.64-39.19) | 26.95 (24.35-29.72) | 29.57 (26.67-32.65) | 88.73 (86.66-90.51) |
| No | 52.28 (51.18-53.38) | 35.29 (32.63-38.05) | 59.39 (56.30-62.41) | 58.25 (55.37-61.06) | 70.05 (67.20-72.75) | 64.82 (61.60-67.92) | 8.96 (7.37-10.86) |
| Don't know | 2.36 (1.97-2.83) | 2.10 (1.42-3.09) | 5.81 (4.52-7.44) | 2.78 (1.97-3.90) | 1.25 (0.71-2.22) | 3.77 (2.59-5.44) | 0.48 (0.20-1.15) |
| Missing | 2.29 (2.04-2.57) | 2.35 (1.63-3.39) | 2.69 (1.81-3.97) | 2.60 (1.83-3.70) | 1.74 (1.11-2.73) | 1.84 (1.17-2.89) | 1.83 (1.17-2.85) |
| **Believed to have had COVID-19 - % (95% CI)** |  |  |  |  |  |  |  |
| Yes | 15.98 (15.31-16.68) | 43.61 (40.82-46.45) | 10.51 (8.74-12.60) | 12.76 (10.95-14.81) | 14.76 (12.68-17.11) | 16.04 (13.82-18.54) | 18.79 (16.52-21.28) |
| No | 69.42 (68.49-70.34) | 49.92 (47.08-52.75) | 68.30 (65.32-71.13) | 63.63 (60.81-66.36) | 68.50 (65.60-71.26) | 72.99 (69.98-75.79) | 62.14 (59.15-65.04) |
| Don't know | 10.75 (10.12-11.42) | 0.00 (0.00-0.00) | 0.00 (0.00-0.00) | 23.61 (21.25-26.15) | 16.74 (14.61-19.12) | 0.00 (0.00-0.00) | 19.08 (16.80-21.58) |
| Missing | 3.84 (3.53-4.18) | 6.47 (5.21-8.02) | 21.19 (18.77-23.84) | 0.00 (0.00-0.00) | 0.00 (0.00-0.00) | 10.97 (9.07-13.21) | 0.00 (0.00-0.00) |
| **Tested positive for COVID-19 - % (95% CI)** |  |  |  |  |  |  |  |
| Yes | 10.53 (9.89-11.22) | 36.22 (33.53-38.99) | 7.89 (6.38-9.72) | 8.07 (6.63-9.79) | 4.57 (3.45-6.02) | 10.26 (8.56-12.25) | 6.45 (5.11-8.12) |
| No | 87.65 (86.94-88.33) | 61.60 (58.80-64.32) | 89.44 (87.37-91.20) | 90.71 (88.89-92.26) | 93.31 (91.64-94.67) | 86.36 (84.04-88.40) | 92.68 (90.93-94.11) |
| Don't know | 0.87 (0.71-1.06) | 0.00 (0.00-0.00) | 0.00 (0.00-0.00) | 1.22 (0.72-2.04) | 2.12 (1.42-3.15) | 0.00 (0.00-0.00) | 0.87 (0.45-1.66) |
| Missing | 0.95 (0.79-1.13) | 2.18 (1.49-3.19) | 2.67 (1.82-3.91) | 0.00 (0.00-0.00) | 0.00 (0.00-0.00) | 3.38 (2.31-4.91) | 0.00 (0.00-0.00) |
| **Relative infected with COVID-19 - % (95% CI)** |  |  |  |  |  |  |  |
| Yes | 30.07 (29.01-31.14) | 57.90 (55.07-60.68) | 20.96 (18.52-23.63) | 32.99 (30.33-35.76) | 24.74 (22.19-27.47) | 28.74 (25.92-31.73) | 28.52 (25.85-31.34) |
| No | 64.92 (63.83-65.99) | 38.66 (35.93-41.46) | 73.88 (71.03-76.54) | 62.50 (59.67-65.25) | 69.44 (66.56-72.17) | 65.25 (62.06-68.30) | 61.95 (58.95-64.85) |
| Don't know | 3.37 (3.02-3.76) | 0.00 (0.00-0.00) | 0.00 (0.00-0.00) | 4.51 (3.46-5.88) | 5.83 (4.56-7.41) | 0.00 (0.00-0.00) | 9.54 (7.89-11.48) |
| Missing | 1.65 (1.44-1.88) | 3.45 (2.55-4.65) | 5.16 (3.94-6.73) | 0.00 (0.00-0.00) | 0.00 (0.00-0.00) | 6.02 (4.53-7.95) | 0.00 (0.00-0.00) |
| **Friend/colleague infected with COVID-19 - % (95% CI)** |  |  |  |  |  |  |  |
| Yes | 42.37 (41.33-43.42) | 62.61 (59.82-65.31) | 46.98 (43.85-50.13) | 54.95 (52.06-57.80) | 41.24 (38.28-44.27) | 40.34 (37.13-43.62) | 50.48 (47.44-53.52) |
| No | 51.75 (50.67-52.83) | 33.95 (31.31-36.69) | 46.27 (43.14-49.44) | 37.50 (34.75-40.33) | 52.49 (49.46-55.50) | 52.76 (49.42-56.08) | 41.23 (38.27-44.26) |
| Don't know | 3.93 (3.59-4.32) | 0.00 (0.00-0.00) | 0.00 (0.00-0.00) | 7.55 (6.16-9.23) | 6.27 (4.96-7.88) | 0.00 (0.00-0.00) | 8.29 (6.76-10.12) |
| Missing | 1.95 (1.72-2.20) | 3.45 (2.55-4.65) | 6.75 (5.31-8.54) | 0.00 (0.00-0.00) | 0.00 (0.00-0.00) | 6.90 (5.30-8.95) | 0.00 (0.00-0.00) |
| **Know of someone dead from COVID-19 - % (95% CI)** |  |  |  |  |  |  |  |
| Yes | 37.02 (35.94-38.10) | 60.59 (57.78-63.33) | 34.42 (31.49-37.47) | 47.92 (45.04-50.81) | 24.87 (22.34-27.58) | 26.56 (23.87-29.43) | 69.85 (66.98-72.56) |
| No | 59.77 (58.68-60.85) | 34.71 (32.05-37.46) | 60.57 (57.45-63.61) | 49.74 (46.86-52.62) | 70.84 (68.01-73.52) | 67.54 (64.43-70.50) | 27.55 (24.92-30.35) |
| Don't know | 1.58 (1.37-1.81) | 0.00 (0.00-0.00) | 0.00 (0.00-0.00) | 2.34 (1.61-3.40) | 4.29 (3.21-5.73) | 0.00 (0.00-0.00) | 2.60 (1.79-3.77) |
| Missing | 1.64 (1.43-1.87) | 4.71 (3.64-6.07) | 5.01 (3.81-6.56) | 0.00 (0.00-0.00) | 0.00 (0.00-0.00) | 5.90 (4.42-7.83) | 0.00 (0.00-0.00) |
| **Comorbidities - % (95% CI)** |  |  |  |  |  |  |  |
| Diabetes | 11.73 (10.84-12.67) | 31.33 (28.71-34.07) | 8.40 (6.64-10.57) | 7.35 (5.97-9.03) | 9.78 (8.21-11.61) | 17.79 (15.42-20.44) | 1.82 (1.15-2.86) |
| Hypertension | 18.94 (17.97-19.95) | 19.37 (17.19-21.76) | 21.21 (18.58-24.10) | 16.30 (14.26-18.57) | 19.19 (17.00-21.58) | 29.56 (26.56-32.75) | 4.34 (3.23-5.80) |
| Heart disease | 4.50 (4.11-4.92) | 13.53 (11.66-15.63) | 4.77 (3.50-6.46) | 3.81 (2.84-5.10) | 4.79 (3.68-6.22) | 6.63 (5.08-8.60) | 1.61 (0.99-2.62) |
| Asthma | 8.44 (7.90-9.00) | 8.81 (7.30-10.60) | 5.95 (4.58-7.69) | 8.33 (6.85-10.09) | 12.91 (10.99-15.11) | 14.47 (12.17-17.12) | 4.44 (3.32-5.91) |
| Allergies^+^ | 18.17 (17.39-18.98) | 15.10 (13.14-17.29) | 16.32 (14.10-18.82) | 19.93 (17.70-22.36) | 13.90 (11.87-16.21) | 27.65 (24.72-30.79) | 26.64 (23.98-29.48) |
| Kidney disease | 2.16 (1.77-2.63) | 5.76 (4.55-7.27) | 1.74 (1.02-2.96) | 1.86 (1.22-2.84) | 1.06 (0.61-1.85) | 2.85 (1.91-4.24) | 1.11 (0.62-1.99) |
| Other condition | 9.01 (8.37-9.69) | 5.24 (4.09-6.69) | 9.81 (8.01-11.96) | 12.05 (10.27-14.08) | 11.49 (9.73-13.53) | 15.80 (13.38-18.56) | 4.34 (3.23-5.80) |
| No comorbidity | 49.55 (48.45-50.66) | 44.07 (41.21-46.96) | 51.68 (48.45-54.89) | 49.60 (46.69-52.52) | 53.98 (50.93-57.01) | 35.86 (32.66-39.20) | 62.56 (59.50-65.52) |

N=sample size; %=weighted percentage; Mean=weighted mean; SD=weighted standard deviation**.**
