# Supplementary material for: The COVID-19 pandemic and health-related quality of life across 13 high- and low-middle-income countries: A cross-sectional analysis
Source: PLoS Med. 2023 Apr 11;20(4):e1004146. doi: 10.1371/journal.pmed.1004146 (PMC10089360; doi:10.1371/journal.pmed.1004146)
Supplement: S3 Table — (DOCX) [file pmed.1004146.s003.docx]

**S3 Table. Indicators of Government responsiveness and perceived effectiveness by country, mean (95% confidence interval)**

| **Country** | **Stringency** | **Government**  **Response** | **Containment Health** | **Economic Support** | **Daily cases** | **Daily deaths** | **Government**  **Effectiveness** |
| --- | --- | --- | --- | --- | --- | --- | --- |
| Australia | 52.65 (52.35-52.94) | 54.63 (54.47-54.79) | 51.72 (51.54-51.90) | 75.00 (75.00-75.00) | 10.92 (10.79-11.05) | 0.04 (0.04-0.04) | 1.57 (1.57-1.57) |
| Brazil | 58.13 (57.92-58.34) | 57.70 (57.58-57.82) | 58.80 (58.66-58.93) | 50.00 (50.00-50.00) | 35356.36 (35050.98-35661.74) | 534.77 (530.34-539.20) | -0.19 (-0.19 - -0.19) |
| Canada | 70.83 (70.83-70.83) | 64.06 (64.06-64.06) | 64.29 (64.29-64.29) | 62.50 (62.50-62.50) | 5647.66 (5634.50-5660.83) | 77.68 (77.41-77.95) | 1.73 (1.73-1.73) |
| Chile | 76.36 (76.16-76.56) | 77.36 (77.19-77.54) | 74.13 (73.94-74.33) | 100.00 (100.00-100.00) | 1471.59 (1412.00-1531.18) | 41.11 (40.91-41.30) | 1.06 (1.06-1.06) |
| China | 79.72 (79.50-79.94) | 74.84 (74.71-74.96) | 76.60 (76.46-76.74) | 62.50 (62.50-62.50) | 13.93 (13.79-14.07) | 0.00 (0.00-0.00) | 0.52 (0.52-0.52) |
| Colombia | 61.93 (61.79-62.06) | 58.27 (58.19-58.35) | 55.88 (55.79-55.96) | 75.00 (75.00-75.00) | 9347.02 (9236.26-9457.78) | 191.34 (189.98-192.70) | 0.07 (0.07-0.07) |
| France | 75.42 (75.15-75.68) | 70.81 (70.66-70.96) | 73.78 (73.61-73.95) | 50.00 (50.00-50.00) | 11879.43 (11822.37-11936.49) | 455.32 (451.61-459.03) | 1.38 (1.38-1.38) |
| India | 68.98 (68.98-68.98) | 64.06 (64.06-64.06) | 66.07 (66.07-66.07) | 50.00 (50.00-50.00) | 24708.42 (24622.49-24794.35) | 348.31 (346.93-349.69) | 0.17 (0.17-0.17) |
| Italy | 79.63 (79.63-79.63) | 74.22 (74.22-74.22) | 74.11 (74.11-74.11) | 75.00 (75.00-75.00) | 23405.97 (23110.56-23701.38) | 703.98 (701.49-706.48) | 0.46 (0.46-0.46) |
| Spain | 71.30 (71.30-71.30) | 66.67 (66.67-66.67) | 63.69 (63.69-63.69) | 87.50 (87.50-87.50) | 9023.02 (8971.47-9074.57) | 263.62 (262.10-265.15) | 1.00 (1.00-1.00) |
| UK | 67.63 (67.42-67.83) | 68.38 (68.19-68.57) | 63.86 (63.64-64.09) | 100.00 (100.00-100.00) | 17451.89 (17097.09-17806.68) | 449.03 (447.52-450.53) | 1.44 (1.44-1.44) |
| US | 74.63 (74.57-74.69) | 68.81 (68.78-68.84) | 69.72 (69.68-69.75) | 62.50 (62.50-62.50) | 175444.00 (174573.10-176314.95) | 1845.20 (1829.33-1861.07) | 1.49 (1.49-1.49) |
| Uganda | 48.18 (48.10-48.26) | 38.66 (38.63-38.70) | 44.19 (44.15-44.23) | 0.00 (0.00-0.00) | 417.74 (410.52-424.95) | 2.23 (2.14-2.32) | -0.59 (-0.59 - -0.59) |

Note: seven-day average prior to survey date.

**Procedures and Indexes definitions**

We captured (macro) indicators of government responses at national level by linking our CANDOUR wave 1 data to the OxCGRT and the WGI databases. Variables of interest from the OxCGRT project, available as time-series, were merged into the CANDOUR dataset using the date in which each participant completed the online survey; variables from the WGI database, available at country-level, were instead merged using the country variable. We used the following four composite indices from the OxCGRT databases: the overall Government Response Index (GRI), which captures how the intensity of the governments responses varies over all indicators in the database during the different phases of the pandemic; the Containment and Health Index (CHI), which combines ‘lockdown’ restrictions and closures with measures to limit the spread of the virus, such as testing policy and contact tracing, short term investment in healthcare, and investments in vaccine); the Economic Support Index (ESI), which records measures such as income support and debt relief; and the Stringency Index, which measures the strictness of ‘lockdown style’ policies that mainly restrain people’s activities and movement. As these indices include the same policy indicators but in different combinations, they were used in separate analyses. Each composite index ranges from 1 to 100 with higher values indicating a higher level of government action in the specific policy area of each index, and in our analyses were expressed as quintiles. We also used an indicator of Government Effectiveness (GE) from the WGI database. The indicator reflects perceptions of: the quality of public and civil services and the degree of its independence from political pressures; the quality of policy formulation and implementation; and the credibility of the government's commitment to such policies for each of the 13 countries in our study. The GE indicator ranges from approximately -2.5 (weak) to 2.5 (strong) governance performance.
