# Supplementary material for: The COVID-19 pandemic and health-related quality of life across 13 high- and low-middle-income countries: A cross-sectional analysis
Source: PLoS Med. 2023 Apr 11;20(4):e1004146. doi: 10.1371/journal.pmed.1004146 (PMC10089360; doi:10.1371/journal.pmed.1004146)
Supplement: S4 Table — (DOCX) [file pmed.1004146.s004.docx]

**S4 Table. Paretian Classification of Health Change overall and by continent, mean (95% confidence interval)**

|  | **Pooled** | **Africa** | **Asia** | **Europe** | **North America** | **Oceania** | **South America** |
| --- | --- | --- | --- | --- | --- | --- | --- |
| No problems | 28.58 (27.6-29.56) | 12.62 (10.6-14.64) | 28.41 (25.39-31.44) | 34.1 (32.66-35.53) | 23.94 (22.06-25.82) | 25.3 (22.85-27.75) | 30.46 (27.85-33.08) |
| No change | 18.6 (17.78-19.43) | 9.34 (7.57-11.12) | 6.15 (4.49-7.82) | 22.63 (21.35-23.91) | 21.81 (19.95-23.68) | 29.97 (27.25-32.69) | 18.45 (16.21-20.69) |
| Improve | 7.2 (6.58-7.82) | 11.37 (9.44-13.3) | 11.82 (9.79-13.84) | 3.91 (3.31-4.5) | 5.03 (4.05-6.01) | 8.2 (6.55-9.86) | 7.95 (6.18-9.71) |
| Worsen | 34.65 (33.6-35.7) | 40.17 (37.19-43.16) | 32.2 (29.41-35) | 34.58 (33.13-36.03) | 39.01 (36.86-41.15) | 25.46 (23.03-27.88) | 35.49 (32.53-38.45) |
| Mixed | 10.96 (10.32-11.6) | 26.49 (23.81-29.18) | 21.41 (18.91-23.92) | 4.79 (4.12-5.45) | 10.21 (8.88-11.54) | 11.07 (9.42-12.72) | 7.65 (6.37-8.92) |
