# Supplementary material for: The COVID-19 pandemic and health-related quality of life across 13 high- and low-middle-income countries: A cross-sectional analysis
Source: PLoS Med. 2023 Apr 11;20(4):e1004146. doi: 10.1371/journal.pmed.1004146 (PMC10089360; doi:10.1371/journal.pmed.1004146)
Supplement: S5 Table — (DOCX) [file pmed.1004146.s005.docx]

**S5 Table. Paretian Classification of Health Change by country, mean (95% confidence interval)**

|  | **Uganda** | **China** | **India** | **France** | **Italy** | **Spain** | **UK** |
| --- | --- | --- | --- | --- | --- | --- | --- |
| No problems | 12.62 (10.6-14.64) | 41.96 (36.62-47.3) | 13.7 (11.74-15.65) | 33.44 (30.53-36.35) | 35.7 (32.69-38.71) | 40.54 (37.7-43.37) | 26.87 (24.21-29.53) |
| No change | 9.34 (7.57-11.12) | 8.03 (5.02-11.04) | 4.12 (2.99-5.25) | 23.19 (20.56-25.82) | 25.18 (22.35-28) | 15.1 (13.04-17.17) | 27.18 (24.53-29.82) |
| Improve | 11.37 (9.44-13.3) | 12.4 (8.88-15.93) | 11.18 (9.39-12.97) | 4.08 (2.78-5.37) | 3.44 (2.32-4.56) | 3.73 (2.64-4.83) | 4.34 (3.1-5.58) |
| Worsen | 40.17 (37.19-43.16) | 25.41 (20.81-30.02) | 39.58 (36.8-42.36) | 32.87 (29.93-35.81) | 30.38 (27.5-33.25) | 38.19 (35.39-41) | 36.59 (33.65-39.53) |
| Mixed | 26.49 (23.81-29.18) | 12.2 (8.06-16.33) | 31.43 (28.79-34.07) | 6.42 (4.81-8.03) | 5.31 (3.94-6.69) | 2.43 (1.54-3.32) | 5.02 (3.65-6.39) |

**S5 Table (Continued). Paretian Classification of Health Change by country, mean (95% confidence interval)**

|  |  | **Canada** | **US** | **Australia** | **Brazil** | **Chile** | **Colombia** |
| --- | --- | --- | --- | --- | --- | --- | --- |
| No problems |  | 23.17 (20.73-25.61) | 24.71 (21.85-27.57) | 25.3 (22.85-27.75) | 29.95 (26.94-32.96) | 23.65 (17.05-30.25) | 37.25 (33.46-41.05) |
| No change |  | 20.64 (18.3-22.99) | 22.99 (20.08-25.9) | 29.97 (27.25-32.69) | 20.57 (17.91-23.24) | 20.18 (14.13-26.23) | 14.43 (11.76-17.1) |
| Improve |  | 4.79 (3.56-6.03) | 5.27 (3.75-6.79) | 8.2 (6.55-9.86) | 6.79 (5.04-8.54) | 6.96 (2.18-11.74) | 10.18 (7.68-12.68) |
| Worsen |  | 45.03 (42.16-47.91) | 32.97 (29.83-36.1) | 25.46 (23.03-27.88) | 31.83 (28.83-34.82) | 46.31 (38.31-54.31) | 29.87 (26.31-33.44) |
| Mixed |  | 6.36 (4.95-7.77) | 14.06 (11.82-16.31) | 11.07 (9.42-12.72) | 10.86 (8.63-13.08) | 2.9 (1.79-4.01) | 8.26 (5.71-10.82) |
