# Supplementary material for: The COVID-19 pandemic and health-related quality of life across 13 high- and low-middle-income countries: A cross-sectional analysis
Source: PLoS Med. 2023 Apr 11;20(4):e1004146. doi: 10.1371/journal.pmed.1004146 (PMC10089360; doi:10.1371/journal.pmed.1004146)
Supplement: S6 Table — (DOCX) [file pmed.1004146.s006.docx]

**S6 Table. Association between worsened health and sociodemographic and clinical characteristics of**

**participants**

|  | **Model 1^a^** | | **Model 2^b^** | | **Model 3^c^** | |
| --- | --- | --- | --- | --- | --- | --- |
|  | *Male* | *Female & Other* | *Male* | *Female & Other* | *Male* | *Female & Other* |
| *Education* |  |  |  |  |  |  |
| Primary or less completed^d^ | - | - | - | - | - | - |
| Secondary completed | 1.144 [0.874,1.497] | 1.713^***^ [1.348,2.176] | 1.030 [0.771,1.376] | 1.511^***^ [1.172,1.949] | 1.141 [0.880,1.480] | 1.581^***^ [1.236,2.022] |
| University completed | 1.433^***^ [1.097,1.871] | 2.461^***^ [1.939,3.122] | 1.226 [0.938,1.602] | 2.008^***^ [1.551,2.600] | 1.372^***^ [1.079,1.744] | 2.160^***^ [1.683,2.773] |
| Missing | 0.887 [0.562,1.401] | 1.526^**^ [1.039,2.241] | 0.808 [0.504,1.296] | 1.200 [0.804,1.789] | 0.959 [0.601,1.533] | 1.484 [0.992,2.219] |
| *Employment* |  |  |  |  |  |  |
| Employed^d^ | - | - | - | - |  | **-** |
| Unemployed | 1.182 [0.980,1.426] | 1.118 [0.933,1.340] | 1.106 [0.910,1.343] | 1.035 [0.857,1.248] |  | 1.064 [0.875,1.293] |
| Pension/Capital Income | 0.944 [0.665,1.342] | 0.802^**^ [0.644,0.999] | 1.218 [0.881,1.683] | 0.919 [0.697,1.212] |  | 0.982 [0.741,1.301] |
| Other | 0.865 [0.719,1.040] | 0.877 [0.740,1.038] | 0.848 [0.683,1.051] | 0.874 [0.734,1.041] |  | 0.960 [0.808,1.142] |
| Missing | 0.840 [0.701,1.006] | 0.799^**^ [0.668,0.955] | 0.770 [0.521,1.139] | 0.537^***^ [0.393,0.733] |  | 0.599^***^ [0.432,0.831] |
| *Income loss* |  |  |  |  |  |  |
| No^d^ | - | - | - | - | - | - |
| Yes | 1.733^***^ [1.501,2.001] | 1.490^***^ [1.313,1.690] | 1.603^***^ [1.377,1.867] | 1.466^***^ [1.282,1.677] | 1.593^***^ [1.378,1.841] | 1.465^***^ [1.284,1.672] |
| Don't know | 0.825 [0.546,1.248] | 0.749 [0.453,1.240] | 0.803 [0.526,1.224] | 0.671 [0.403,1.118] | 0.857 [0.557,1.319] | 0.796 [0.509,1.243] |
| Missing | 1.431 [0.991,2.068] | 1.041 [0.751,1.444] | 1.356 [0.939,1.958] | 0.978 [0.700,1.366] | 1.512^**^ [1.038,2.202] | 1.055 [0.744,1.496] |
| *Health conditions* |  |  |  |  |  |  |
| 0^d^ | - | - | - | - | - | - |
| 1 | 1.121 [0.984,1.278] | 1.296^***^ [1.124,1.494] | 1.191^**^ [1.040,1.363] | 1.416^***^ [1.220,1.644] | 1.179^**^ [1.027,1.355] | 1.460^***^ [1.267,1.682] |
| 2+ | 1.580^***^ [1.204,2.072] | 1.434^***^ [1.220,1.685] | 1.720^***^ [1.374,2.154] | 1.658^***^ [1.393,1.974] | 1.696^***^ [1.371,2.098] | 1.683^***^ [1.412,2.005] |
| Missing | 0.829 [0.585,1.174] | 1.084 [0.775,1.517] | 0.769 [0.505,1.173] | 1.033 [0.734,1.454] | 0.786 [0.512,1.205] | 1.260 [0.883,1.799] |

OR [95% confidence interval]; ^a^Unadjusted; ^b^Adjusted by age and country; ^c^Fully adjusted; ^d^Reference category; ** p < 0.05; *** p < 0.01.
