# Supplementary material for: The COVID-19 pandemic and health-related quality of life across 13 high- and low-middle-income countries: A cross-sectional analysis
Source: PLoS Med. 2023 Apr 11;20(4):e1004146. doi: 10.1371/journal.pmed.1004146 (PMC10089360; doi:10.1371/journal.pmed.1004146)
Supplement: S7 Table — (DOCX) [file pmed.1004146.s007.docx]

**S7 Table. Association between worsened health and experiences of/exposure to COVID-19**

|  | **Model 1^a^** | | **Model 2^b^** | | **Model 3^c^** | |
| --- | --- | --- | --- | --- | --- | --- |
|  | *Male* | *Female & Other* | *Male* | *Female & Other* | *Male* | *Female & Other* |
| *Believed had COVID-19* |  |  |  |  |  |  |
| No^d^ | - | - | - | - | - | - |
| Yes | 1.087 [0.931,1.269] | 1.358^***^ [1.160,1.590] | 0.993 [0.853,1.156] | 1.266^***^ [1.069,1.500] | 0.895 [0.747,1.074] | 1.274^***^ [1.063,1.526] |
| Don't know | 1.394^***^ [1.148,1.693] | 1.409^***^ [1.142,1.739] | 1.307^**^ [1.052,1.624] | 1.226 [0.986,1.523] | 1.277^**^ [1.015,1.606] | 1.225 [0.988,1.520] |
| Missing | 0.796 [0.587,1.079] | 1.061 [0.830,1.356] | 0.859 [0.627,1.176] | 1.146 [0.882,1.491] | 0.913 [0.649,1.283] | 1.259 [0.944,1.678] |
| *Tested positive for COVID-19* |  |  |  |  |  |  |
| No^d^ | - | - | - | - | **-** | - |
| Yes | 1.021 [0.847,1.232] | 0.903 [0.733,1.111] | 0.936 [0.757,1.158] | 0.915 [0.733,1.143] | 0.866 [0.678,1.105] | 0.776^**^ [0.614,0.980] |
| Don't know | 0.860 [0.436,1.696] | 0.503 [0.251,1.010] | 0.814 [0.390,1.699] | 0.447^**^ [0.219,0.914] | 0.887 [0.399,1.973] | 0.465^**^ [0.220,0.982] |
| Missing | 0.450^**^ [0.232,0.875] | 0.628 [0.333,1.184] | 0.467^**^ [0.235,0.928] | 0.657 [0.351,1.230] | 0.462 [0.205,1.044] | 0.831 [0.356,1.938] |
| *Relative had COVID-19* |  |  |  |  |  |  |
| No^d^ | - | - | - | - | - | - |
| Yes | 1.395^***^ [1.174,1.658] | 1.175^**^ [1.023,1.348] | 1.335^***^ [1.133,1.573] | 1.073 [0.925,1.246] | 1.245^**^ [1.026,1.511] | 0.926 [0.795,1.078] |
| Don't know | 1.079 [0.803,1.450] | 1.006 [0.697,1.451] | 0.984 [0.722,1.340] | 0.847 [0.580,1.238] | 0.932 [0.636,1.365] | 0.767 [0.490,1.201] |
| Missing | 0.699 [0.445,1.098] | 0.600^**^ [0.367,0.980] | 0.769 [0.481,1.230] | 0.606^**^ [0.376,0.977] | 0.885 [0.498,1.571] | 0.573 [0.283,1.157] |
| *Friend/colleague had COVID-19* |  |  |  |  |  |  |
| No^d^ | - | - | - | - | - | - |
| Yes | 1.361^***^ [1.171,1.581] | 1.477^***^ [1.303,1.673] | 1.319^***^ [1.133,1.536] | 1.397^***^ [1.211,1.611] | 1.216^**^ [1.010,1.464] | 1.338^***^ [1.161,1.543] |
| Don't know | 1.229 [0.904,1.671] | 1.448^**^ [1.083,1.937] | 1.137 [0.819,1.578] | 1.238 [0.908,1.690] | 1.198 [0.823,1.743] | 1.377 [0.972,1.950] |
| Missing | 0.812 [0.541,1.218] | 0.877 [0.577,1.332] | 0.911 [0.605,1.371] | 0.902 [0.593,1.370] | 0.964 [0.579,1.603] | 1.131 [0.660,1.939] |
| *Known death from COVID-19* |  |  |  |  |  |  |
| No^d^ | - | - | - | - | - | - |
| Yes | 1.417^***^ [1.216,1.650] | 1.360^***^ [1.198,1.544] | 1.329^***^ [1.144,1.544] | 1.354^***^ [1.169,1.570] | 1.229^**^ [1.045,1.446] | 1.270^***^ [1.095,1.473] |
| Don't know | 0.804 [0.525,1.232] | 0.998 [0.649,1.534] | 0.725 [0.466,1.127] | 0.850 [0.544,1.327] | 0.671 [0.386,1.165] | 0.902 [0.558,1.457] |
| Missing | 1.083 [0.726,1.615] | 0.811 [0.517,1.272] | 1.111 [0.737,1.675] | 0.803 [0.517,1.247] | 1.569 [0.941,2.614] | 1.007 [0.587,1.728] |

OR [95% confidence interval]; ^a^Unadjusted; ^b^Adjusted by age and country; ^c^Fully adjusted; ^d^Reference category; ** p < 0.05; *** p < 0.01.
