# Supplementary material for: The COVID-19 pandemic and health-related quality of life across 13 high- and low-middle-income countries: A cross-sectional analysis
Source: PLoS Med. 2023 Apr 11;20(4):e1004146. doi: 10.1371/journal.pmed.1004146 (PMC10089360; doi:10.1371/journal.pmed.1004146)
Supplement: S8 Table — (DOCX) [file pmed.1004146.s008.docx]

**S8 Table. Association between worsened health and indicators of**

**responsiveness to and severity of COVID-19**

|  | **Model 1^a^** | | **Model 2^b^** | |
| --- | --- | --- | --- | --- |
|  | *Male* | *Female & Other* | *Male* | *Female & Other* |
| *Stringency Index* |  |  |  |  |
| 1 – Lower quintile^c^ | - | - | - | - |
| 2 | 0.750^***^ [0.627,0.896] | 1.085 [0.910,1.294] | 0.762 [0.548,1.059] | 0.897 [0.657,1.225] |
| 3 | 1.265^***^ [1.096,1.461] | 1.580^***^ [1.347,1.853] | 0.885 [0.583,1.344] | 0.589 [0.260,1.334] |
| 4 | 0.959 [0.791,1.163] | 1.442^***^ [1.165,1.785] | 1.229 [0.461,3.277] | 1.173 [0.715,1.925] |
| 5 – Upper quintile | 0.888 [0.664,1.186] | 0.950 [0.781,1.156] | 2.931^**^ [1.128,7.611] | 1.052 [0.612,1.808] |
| *Gov. Response Index* |  |  |  |  |
| 1 – Lower quintile^c^ | - | - | - | - |
| 2 | 1.073 [0.925,1.245] | 1.255^***^ [1.068,1.474] | 0.765 [0.550,1.064] | 0.897 [0.657,1.225] |
| 3 | 0.962 [0.806,1.149] | 1.332^***^ [1.109,1.599] | 0.810 [0.600,1.095] | 0.901 [0.632,1.284] |
| 4 | 0.765^***^ [0.655,0.894] | 1.237^**^ [1.051,1.456] | 0.680^**^ [0.502,0.923] | 1.414 [0.995,2.011] |
| 5 – Upper quintile | 1.119 [0.790,1.587] | 1.236 [0.949,1.610] | 1.343 [0.609,2.962] | 1.374 [0.848,2.226] |
| *Contain. Health Index* |  |  |  |  |
| 1 – Lower quintile^c^ | - | - | - | - |
| 2 | 0.864 [0.738,1.011] | 1.287^***^ [1.095,1.512] | 0.754 [0.558,1.021] | 0.903 [0.707,1.153] |
| 3 | 1.465^***^ [1.253,1.713] | 1.691^***^ [1.419,2.014] | 1.059 [0.713,1.573] | 1.276 [0.415,3.924] |
| 4 | 0.934 [0.790,1.105] | 1.307^***^ [1.093,1.563] | 1.092 [0.256,4.659] | 1.287 [0.781,2.120] |
| 5 – Upper quintile | 0.966 [0.661,1.411] | 0.979 [0.753,1.273] | 2.684 [0.478,15.06] | 1.540 [0.854,2.778] |
| *Econ. Support Index* |  |  |  |  |
| 1 – Lower quintile^c^ | - | - | - | - |
| 2 | 0.867 [0.733,1.026] | 0.991 [0.841,1.168] | 0.635^***^ [0.487,0.828] | 1.005 [0.729,1.386] |
| 3 | 0.699^***^ [0.597,0.818] | 0.687^***^ [0.593,0.797] | 0.696^**^ [0.527,0.919] | 0.784 [0.575,1.068] |
| 4 | 0.920 [0.755,1.120] | 1.259^**^ [1.040,1.523] | 0.862 [0.665,1.118] | 1.201 [0.892,1.617] |
| 5 – Upper quintile | 1.180 [0.854,1.631] | 1.283^**^ [1.018,1.617] | 0.754^**^ [0.579,0.983] | 1.167 [0.857,1.589] |
| *New cases* |  |  |  |  |
| 1 – Lower quintile^c^ | - | - | - | - |
| 2 | 1.691^***^ [1.319,2.168] | 1.977^***^ [1.586,2.465] | 0.978 [0.730,1.310] | 0.780 [0.481,1.266] |
| 3 | 1.133 [0.915,1.402] | 1.619^***^ [1.325,1.979] | 1.209 [0.741,1.973] | 0.817 [0.462,1.445] |
| 4 | 1.248^**^ [1.033,1.508] | 1.502^***^ [1.246,1.809] | 0.290^**^ [0.0998,0.844] | 0.576 [0.265,1.254] |
| 5 – Upper quintile | 0.963 [0.775,1.197] | 1.563^***^ [1.279,1.910] | 0.446 [0.0770,2.583] | 0.677 [0.271,1.688] |
| *New deaths* |  |  |  |  |
| 1 – Lower quintile^c^ | - | - | - | - |
| 2 | 1.643^***^ [1.279,2.110] | 1.856^***^ [1.490,2.312] | 0.972 [0.722,1.308] | 0.806 [0.497,1.308] |
| 3 | 1.393^***^ [1.148,1.689] | 1.743^***^ [1.437,2.113] | 1.172 [0.630,2.180] | 1.002 [0.539,1.862] |
| 4 | 1.038 [0.849,1.269] | 1.675^***^ [1.376,2.038] | 0.746^**^ [0.557,0.999] | 1.060 [0.725,1.549] |
| 5 – Upper quintile | 0.926 [0.760,1.130] | 1.417^***^ [1.170,1.716] | 0.643^**^ [0.427,0.969] | 0.953 [0.595,1.528] |

OR [95% confidence interval]; ^a^Unadjusted; ^b^Adjusted by age and country; ^c^Reference category;

** p < 0.05; *** p < 0.01.
