# Supplementary material for: The COVID-19 pandemic and health-related quality of life across 13 high- and low-middle-income countries: A cross-sectional analysis
Source: PLoS Med. 2023 Apr 11;20(4):e1004146. doi: 10.1371/journal.pmed.1004146 (PMC10089360; doi:10.1371/journal.pmed.1004146)
Supplement: S9 Table — (DOCX) [file pmed.1004146.s009.docx]

**S9 Table. Association between worsened health and perceived government**

**effectiveness**

|  | **Model 1^a^** | | **Model 2^b^** | |
| --- | --- | --- | --- | --- |
|  | *Male* | *Female & Other* | *Male* | *Female & Other* |
| *Gov. effectiveness* |  |  |  |  |
| 1 – Lower quintile^c^ | - | - | - | - |
| 2 | 0.979 [0.816,1.174] | 0.857 [0.715,1.028] | 0.696^**^ [0.527,0.919] | 0.784 [0.575,1.068] |
| 3 | 1.391 [0.999,1.936] | 1.449^***^ [1.142,1.838] | 0.862 [0.665,1.118] | 1.201 [0.892,1.617] |
| 4 | 0.842^**^ [0.718,0.988] | 1.193^**^ [1.013,1.405] | 0.754^**^ [0.579,0.983] | 1.167 [0.857,1.589] |
| 5 – Upper quintile | 1.038 [0.878,1.227] | 1.025 [0.865,1.215] | 1.229 [0.967,1.561] | 1.528^***^ [1.129,2.069] |

OR [95% confidence interval]; ^a^Unadjusted; ^b^Adjusted by age and country; ^c^Reference category;

** p < 0.05; *** p < 0.01.
