# Supplementary material for: The COVID-19 pandemic and health-related quality of life across 13 high- and low-middle-income countries: A cross-sectional analysis
Source: PLoS Med. 2023 Apr 11;20(4):e1004146. doi: 10.1371/journal.pmed.1004146 (PMC10089360; doi:10.1371/journal.pmed.1004146)
Supplement: S10 Table — (DOCX) [file pmed.1004146.s010.docx]

**S10 Table. Respondents self-reported health on EQ-5D-5L before the COVID-19 pandemic and at time of survey – overall sample**

| EQ-5D Question | Level 1 | | | Level 2 | | | Level 3 | | | Level 4 | | | Level 5 | | |
| --- | --- | --- | --- | --- | --- | --- | --- | --- | --- | --- | --- | --- | --- | --- | --- |
|  | N | % | p-value^a^ | N | % | p-value^a^ | N | % | p-value^a^ | N | % | p-value^a^ | N | % | p-value^a^ |
| Mobility |  |  |  |  |  |  |  |  |  |  |  |  |  |  |  |
| Pre-COVID-19 | 12,374 | 78.35 |  | 1,307 | 9.11 |  | 855 | 5.93 |  | 490 | 3.39 |  | 454 | 3.21 |  |
| During COVID-19 | 11,469 | 72.93^*^ | <0.001 | 1,840 | 12.03^*^ | <0.001 | 1,041 | 7.34^*^ | 0.002 | 646 | 4.38^*^ | <0.001 | 484 | 3.32 | 0.635 |
| Self-care |  |  |  |  |  |  |  |  |  |  |  |  |  |  |  |
| Pre-COVID-19 | 13,467 | 86.01 |  | 991 | 6.92 |  | 479 | 3.47 |  | 242 | 1.59 |  | 301 | 2.01 |  |
| During COVID-19 | 13,084 | 83.78^*^ | <0.001 | 1,056 | 7.37 | 0.148 | 661 | 4.39^*^ | <0.001 | 380 | 2.55^*^ | <0.001 | 299 | 1.91 | 0.453 |
| Usual activity |  |  |  |  |  |  |  |  |  |  |  |  |  |  |  |
| Pre-COVID-19 | 12,787 | 81.13 |  | 1,391 | 9.77 |  | 692 | 5.03 |  | 346 | 2.21 |  | 264 | 1.86 |  |
| During COVID-19 | 11,017 | 70.5^*^ | <0.001 | 2,105 | 13.98^*^ | <0.001 | 1,247 | 8.35^*^ | <0.001 | 664 | 4.27^*^ | <0.001 | 447 | 2.9^*^ | <0.001 |
| Pain/discomfort |  |  |  |  |  |  |  |  |  |  |  |  |  |  |  |
| Pre-COVID-19 | 10,727 | 67.47 |  | 2,592 | 17.68 |  | 1,244 | 8.61 |  | 570 | 3.88 |  | 347 | 2.36 |  |
| During COVID-19 | 9,738 | 62.34^*^ | <0.001 | 2,936 | 18.75 | 0.053 | 1,578 | 10.39^*^ | <0.001 | 777 | 5.56^*^ | <0.001 | 451 | 2.96^*^ | <0.001 |
| Anxiety/depression |  |  |  |  |  |  |  |  |  |  |  |  |  |  |  |
| Pre-COVID-19 | 9,974 | 63.91 |  | 3,207 | 20.62 |  | 1,336 | 8.65 |  | 578 | 4.05 |  | 385 | 2.77 |  |
| During COVID-19 | 7,088 | 46.88^*^ | <0.001 | 3,973 | 25.04^*^ | <0.001 | 2,461 | 15.27^*^ | <0.001 | 1,165 | 7.32^*^ | <0.001 | 793 | 5.49^*^ | <0.001 |

*Sample size:* 15,480 ^a^ p-value referring to the proportion of respondents reporting different level of response at the time of the survey compared with before the

COVID-19 pandemic: ^*^ significant at the 1% level or less; ^**^ significant at the 5% level.
