# Supplementary material for: The COVID-19 pandemic and health-related quality of life across 13 high- and low-middle-income countries: A cross-sectional analysis
Source: PLoS Med. 2023 Apr 11;20(4):e1004146. doi: 10.1371/journal.pmed.1004146 (PMC10089360; doi:10.1371/journal.pmed.1004146)
Supplement: S11 Table — (DOCX) [file pmed.1004146.s011.docx]

**S11 Table. Mean difference in EQ-5D-5L index (utility) pre-COVID-19 and at**

**time of survey, UK value set – Male only**

| Country | Utility pre-COVID-19 | | | Utility at survey | | | Utility difference | | |
| --- | --- | --- | --- | --- | --- | --- | --- | --- | --- |
|  | N | Mean | SD | N | Mean | SD | Mean | 95% CI | p-value |
| Australia | 642 | 0.766 | 0.262 | 642 | 0.709 | 0.293 | -0.056 | (-0.089, -0.024) | 0.001 |
| Brazil | 706 | 0.836 | 0.248 | 706 | 0.797 | 0.269 | -0.039 | (-0.075, -0.003) | 0.033 |
| Canada | 617 | 0.814 | 0.236 | 617 | 0.741 | 0.285 | -0.072 | (-0.102, -0.043) | <0.001 |
| Chile | 436 | 0.865 | 0.238 | 436 | 0.718 | 0.332 | -0.147 | (-0.317, 0.023) | 0.09 |
| China | 683 | 0.876 | 0.208 | 683 | 0.881 | 0.158 | 0.006 | (-0.032, 0.043) | 0.767 |
| Colombia | 520 | 0.849 | 0.271 | 520 | 0.849 | 0.236 | -0.001 | (-0.054, 0.053) | 0.977 |
| France | 634 | 0.860 | 0.219 | 634 | 0.821 | 0.233 | -0.039 | (-0.065, -0.013) | 0.004 |
| India | 720 | 0.721 | 0.348 | 720 | 0.605 | 0.349 | -0.116 | (-0.152, -0.080) | <0.001 |
| Italy | 488 | 0.871 | 0.207 | 488 | 0.827 | 0.244 | -0.044 | (-0.074, -0.014) | 0.004 |
| Spain | 560 | 0.916 | 0.157 | 560 | 0.878 | 0.174 | -0.038 | (-0.057, -0.019) | <0.001 |
| UK | 625 | 0.809 | 0.271 | 625 | 0.773 | 0.277 | -0.036 | (-0.068, -0.004) | 0.027 |
| US | 580 | 0.739 | 0.310 | 580 | 0.670 | 0.346 | -0.069 | (-0.114, -0.024) | 0.003 |
| Uganda | 762 | 0.737 | 0.346 | 762 | 0.573 | 0.405 | -0.164 | (-0.202, -0.127) | <0.001 |
| Overall | 7,973 | 0.816 | 0.271 | 7,973 | 0.753 | 0.304 | -0.063 | (-0.078, -0.049) | <0.001 |

N=sample size; Mean=weighted mean; SD=weighted standard deviation; CI=confidence interval.
