# Supplementary material for: The COVID-19 pandemic and health-related quality of life across 13 high- and low-middle-income countries: A cross-sectional analysis
Source: PLoS Med. 2023 Apr 11;20(4):e1004146. doi: 10.1371/journal.pmed.1004146 (PMC10089360; doi:10.1371/journal.pmed.1004146)
Supplement: S12 Table — (DOCX) [file pmed.1004146.s012.docx]

**S12 Table. Mean difference in EQ-5D-5L index (utility) pre-COVID-19 and at**

**time of survey, UK value set – Female and Other only**

| Country | Utility pre-COVID-19 | | | Utility at survey | | | Utility difference | | |
| --- | --- | --- | --- | --- | --- | --- | --- | --- | --- |
|  | N | Mean | SD | N | Mean | SD | Mean | 95% CI | p-value |
| Australia | 716 | 0.777 | 0.260 | 716 | 0.726 | 0.292 | -0.050 | (-0.083, -0.018) | 0.002 |
| Brazil | 715 | 0.829 | 0.220 | 715 | 0.745 | 0.268 | -0.083 | (-0.115, -0.051) | <0.001 |
| Canada | 531 | 0.812 | 0.225 | 531 | 0.720 | 0.270 | -0.092 | (-0.122, -0.062) | <0.001 |
| Chile | 684 | 0.848 | 0.208 | 684 | 0.768 | 0.230 | -0.080 | (-0.121, -0.039) | <0.001 |
| China | 608 | 0.884 | 0.175 | 608 | 0.868 | 0.211 | -0.016 | (-0.063, 0.031) | 0.498 |
| Colombia | 711 | 0.867 | 0.223 | 711 | 0.815 | 0.241 | -0.052 | (-0.085, -0.020) | 0.001 |
| France | 508 | 0.832 | 0.245 | 508 | 0.781 | 0.261 | -0.052 | (-0.086, -0.018) | 0.003 |
| India | 470 | 0.688 | 0.358 | 470 | 0.591 | 0.372 | -0.097 | (-0.143, -0.050) | <0.001 |
| Italy | 592 | 0.847 | 0.190 | 592 | 0.791 | 0.233 | -0.056 | (-0.082, -0.031) | <0.001 |
| Spain | 592 | 0.888 | 0.190 | 592 | 0.826 | 0.205 | -0.062 | (-0.085, -0.040) | <0.001 |
| UK | 538 | 0.798 | 0.259 | 538 | 0.728 | 0.283 | -0.070 | (-0.104, -0.037) | <0.001 |
| US | 566 | 0.768 | 0.261 | 566 | 0.683 | 0.311 | -0.085 | (-0.124, -0.046) | <0.001 |
| Uganda | 276 | 0.710 | 0.386 | 276 | 0.563 | 0.406 | -0.148 | (-0.214, -0.082) | <0.001 |
| Overall | 7,507 | 0.817 | 0.250 | 7,507 | 0.748 | 0.283 | -0.069 | (-0.079, -0.059) | <0.001 |

N=sample size; Mean=weighted mean; SD=weighted standard deviation; CI=confidence interval.
