# Supplementary material for: The COVID-19 pandemic and health-related quality of life across 13 high- and low-middle-income countries: A cross-sectional analysis
Source: PLoS Med. 2023 Apr 11;20(4):e1004146. doi: 10.1371/journal.pmed.1004146 (PMC10089360; doi:10.1371/journal.pmed.1004146)
Supplement: S13 Table — (DOCX) [file pmed.1004146.s013.docx]

**S13 Table. Mean difference in EQ-5D-5L index (utility) pre-COVID-19 and at time of survey,**

**US value set – overall sample**

| **Country** | **EQ-5D index pre-COVID-19** | | | **EQ-5D index at survey** | | | **EQ-5D index at survey –**  **EQ-5D index pre-pandemic** | | |
| --- | --- | --- | --- | --- | --- | --- | --- | --- | --- |
|  | **N** | **Mean** | **SD** | **N** | **Mean** | **SD** | **Mean difference** | **95% CI** | **p-value** |
| Australia | 1,358 | 0.801 | 0.269 | 1,358 | 0.749 | 0.308 | -0.053 | (-0.076, -0.029) | <0.001 |
| Brazil | 1,421 | 0.857 | 0.239 | 1,421 | 0.803 | 0.274 | -0.054 | (-0.079, -0.029) | <0.001 |
| Canada | 1,148 | 0.844 | 0.234 | 1,148 | 0.763 | 0.290 | -0.082 | (-0.103, -0.060) | <0.001 |
| Chile | 1,120 | 0.888 | 0.222 | 1,120 | 0.784 | 0.273 | -0.103 | (-0.177, -0.030) | 0.006 |
| China | 1,291 | 0.890 | 0.205 | 1,291 | 0.891 | 0.188 | 0.001 | (-0.031, 0.033) | 0.935 |
| Colombia | 1,231 | 0.878 | 0.249 | 1,231 | 0.857 | 0.233 | -0.021 | (-0.051, 0.009) | 0.177 |
| France | 1,142 | 0.875 | 0.236 | 1,142 | 0.835 | 0.255 | -0.039 | (-0.062, -0.017) | 0.001 |
| India | 1,190 | 0.711 | 0.373 | 1,190 | 0.595 | 0.390 | -0.116 | (-0.146, -0.085) | <0.001 |
| Italy | 1,080 | 0.889 | 0.200 | 1,080 | 0.844 | 0.243 | -0.045 | (-0.065, -0.025) | <0.001 |
| Spain | 1,152 | 0.930 | 0.170 | 1,152 | 0.888 | 0.188 | -0.042 | (-0.056, -0.027) | <0.001 |
| UK | 1,163 | 0.837 | 0.266 | 1,163 | 0.784 | 0.291 | -0.053 | (-0.077, -0.030) | <0.001 |
| US | 1,146 | 0.780 | 0.299 | 1,146 | 0.699 | 0.351 | -0.082 | (-0.113, -0.050) | <0.001 |
| Uganda | 1,038 | 0.733 | 0.368 | 1,038 | 0.565 | 0.421 | -0.167 | (-0.201, -0.133) | <0.001 |
| *Overall* | 15,480 | 0.840 | 0.269 | 15,480 | 0.776 | 0.306 | -0.064 | (-0.073, -0.055) | <0.001 |

N=sample size; Mean=weighted mean; SD=weighted standard deviation; CI=confidence interval.
