# Supplementary material for: The COVID-19 pandemic and health-related quality of life across 13 high- and low-middle-income countries: A cross-sectional analysis
Source: PLoS Med. 2023 Apr 11;20(4):e1004146. doi: 10.1371/journal.pmed.1004146 (PMC10089360; doi:10.1371/journal.pmed.1004146)
Supplement: S14 Table — (DOCX) [file pmed.1004146.s014.docx]

**S14 Table. Mean difference in EQ-5D-5L index (utility) pre-COVID-19 and at time of survey,**

**US value set – Male only**

|  | **EQ-5D index pre-COVID-19** | | | **EQ-5D index at survey** | | | **EQ-5D index at survey –**  **EQ-5D index pre-pandemic** | | |
| --- | --- | --- | --- | --- | --- | --- | --- | --- | --- |
| **Country** | **N** | **Mean** | **SD** | **N** | **Mean** | **SD** | **Mean difference** | **95% CI** | **p-value** |
| Australia | 642 | 0.787 | 0.280 | 642 | 0.729 | 0.316 | -0.058 | (-0.093, -0.023) | 0.001 |
| Brazil | 706 | 0.851 | 0.258 | 706 | 0.821 | 0.279 | -0.030 | (-0.068, 0.008) | 0.120 |
| Canada | 617 | 0.843 | 0.240 | 617 | 0.768 | 0.297 | -0.075 | (-0.105, -0.045) | <0.001 |
| Chile | 436 | 0.891 | 0.248 | 436 | 0.750 | 0.321 | -0.141 | (-0.301, 0.020) | 0.086 |
| China | 683 | 0.884 | 0.220 | 683 | 0.896 | 0.159 | 0.012 | (-0.030, 0.054) | 0.569 |
| Colombia | 520 | 0.860 | 0.282 | 520 | 0.871 | 0.235 | 0.011 | (-0.046, 0.067) | 0.713 |
| France | 634 | 0.887 | 0.220 | 634 | 0.856 | 0.233 | -0.031 | (-0.058, -0.005) | 0.018 |
| India | 720 | 0.721 | 0.370 | 720 | 0.596 | 0.383 | -0.125 | (-0.164, -0.086) | <0.001 |
| Italy | 488 | 0.896 | 0.211 | 488 | 0.854 | 0.253 | -0.041 | (-0.073, -0.010) | 0.010 |
| Spain | 560 | 0.940 | 0.157 | 560 | 0.908 | 0.176 | -0.032 | (-0.052, -0.013) | 0.001 |
| UK | 625 | 0.839 | 0.270 | 625 | 0.803 | 0.284 | -0.036 | (-0.068, -0.004) | 0.027 |
| US | 580 | 0.754 | 0.329 | 580 | 0.682 | 0.368 | -0.072 | (-0.119, -0.025) | 0.003 |
| Uganda | 762 | 0.737 | 0.356 | 762 | 0.566 | 0.423 | -0.171 | (-0.211, -0.132) | <0.001 |
| *Overall* | 7,973 | 0.833 | 0.283 | 7,973 | 0.771 | 0.319 | -0.062 | (-0.076, -0.047) | <0.001 |

N=sample size; Mean=weighted mean; SD=weighted standard deviation; CI=confidence interval.
