# Supplementary material for: The COVID-19 pandemic and health-related quality of life across 13 high- and low-middle-income countries: A cross-sectional analysis
Source: PLoS Med. 2023 Apr 11;20(4):e1004146. doi: 10.1371/journal.pmed.1004146 (PMC10089360; doi:10.1371/journal.pmed.1004146)
Supplement: S15 Table — (DOCX) [file pmed.1004146.s015.docx]

**S15 Table. Mean difference in EQ-5D-5L index (utility) pre-COVID-19 and at time of**

**survey, US value set – Female and Other only**

|  | **EQ-5D index pre-COVID-19** | | | **EQ-5D index at survey** | | | **EQ-5D index at survey –**  **EQ-5D index pre-pandemic** | | |
| --- | --- | --- | --- | --- | --- | --- | --- | --- | --- |
| **Country** | **N** | **Mean** | **SD** | **N** | **Mean** | **SD** | **Mean** | **95% CI** | **p-value** |
| Australia | 716 | 0.814 | 0.259 | 716 | 0.765 | 0.299 | -0.048 | (-0.081, -0.016) | 0.004 |
| Brazil | 715 | 0.862 | 0.220 | 715 | 0.785 | 0.268 | -0.077 | (-0.109, -0.045) | <0.001 |
| Canada | 531 | 0.846 | 0.228 | 531 | 0.757 | 0.281 | -0.089 | (-0.120, -0.058) | <0.001 |
| Chile | 684 | 0.885 | 0.202 | 684 | 0.809 | 0.230 | -0.077 | (-0.115, -0.039) | <0.001 |
| China | 608 | 0.899 | 0.179 | 608 | 0.883 | 0.226 | -0.016 | (-0.066, 0.034) | 0.535 |
| Colombia | 711 | 0.892 | 0.218 | 711 | 0.846 | 0.231 | -0.045 | (-0.077, -0.014) | 0.004 |
| France | 508 | 0.864 | 0.250 | 508 | 0.817 | 0.272 | -0.047 | (-0.082, -0.011) | 0.01 |
| India | 470 | 0.696 | 0.377 | 470 | 0.593 | 0.401 | -0.102 | (-0.152, -0.053) | <0.001 |
| Italy | 592 | 0.884 | 0.190 | 592 | 0.836 | 0.233 | -0.048 | (-0.074, -0.022) | <0.001 |
| Spain | 592 | 0.921 | 0.182 | 592 | 0.870 | 0.198 | -0.051 | (-0.072, -0.029) | <0.001 |
| UK | 538 | 0.835 | 0.263 | 538 | 0.764 | 0.296 | -0.071 | (-0.105, -0.036) | <0.001 |
| US | 566 | 0.806 | 0.265 | 566 | 0.715 | 0.333 | -0.091 | (-0.132, -0.050) | <0.001 |
| Uganda | 276 | 0.720 | 0.399 | 276 | 0.564 | 0.416 | -0.156 | (-0.224, -0.088) | <0.001 |
| *Overall* | 7,507 | 0.848 | 0.253 | 7,507 | 0.781 | 0.292 | -0.066 | (-0.077, -0.056) | <0.001 |

N= sample size; Mean=weighted mean; SD=weighted standard deviation; CI=confidence interval.
