# Supplementary material for: The COVID-19 pandemic and health-related quality of life across 13 high- and low-middle-income countries: A cross-sectional analysis
Source: PLoS Med. 2023 Apr 11;20(4):e1004146. doi: 10.1371/journal.pmed.1004146 (PMC10089360; doi:10.1371/journal.pmed.1004146)
Supplement: S16 Table — (DOCX) [file pmed.1004146.s016.docx]

**S16 Table. Mean difference in EQ-5D-5L index (utility) pre-COVID-19 and at time of survey,**

**India value set – overall sample**

| **Country** | **EQ-5D index pre-COVID-19** | | | **EQ-5D index at survey** | | | **EQ-5D index at survey –**  **EQ-5D index pre-pandemic** | | |
| --- | --- | --- | --- | --- | --- | --- | --- | --- | --- |
|  | **N** | **Mean** | **SD** | **N** | **Mean** | **SD** | **Mean difference** | **95% CI** | **p-value** |
| Australia | 1,358 | 0.826 | 0.280 | 1,358 | 0.778 | 0.322 | -0.048 | (-0.073, -0.023) | <0.001 |
| Brazil | 1,421 | 0.876 | 0.248 | 1,421 | 0.834 | 0.278 | -0.043 | (-0.068, -0.017) | 0.001 |
| Canada | 1,148 | 0.867 | 0.242 | 1,148 | 0.800 | 0.300 | -0.067 | (-0.089, -0.045) | <0.001 |
| Chile | 1,120 | 0.908 | 0.233 | 1,120 | 0.825 | 0.269 | -0.083 | (-0.154, -0.011) | 0.024 |
| China | 1,291 | 0.906 | 0.199 | 1,291 | 0.913 | 0.187 | 0.007 | (-0.025, 0.040) | 0.656 |
| Colombia | 1,231 | 0.892 | 0.269 | 1,231 | 0.880 | 0.248 | -0.012 | (-0.044, 0.020) | 0.469 |
| France | 1,142 | 0.889 | 0.254 | 1,142 | 0.863 | 0.265 | -0.026 | (-0.049, -0.002) | 0.034 |
| India | 1,190 | 0.723 | 0.407 | 1,190 | 0.626 | 0.430 | -0.097 | (-0.131, -0.064) | <0.001 |
| Italy | 1,080 | 0.910 | 0.198 | 1,080 | 0.874 | 0.244 | -0.036 | (-0.056, -0.016) | <0.001 |
| Spain | 1,152 | 0.944 | 0.171 | 1,152 | 0.916 | 0.186 | -0.028 | (-0.042, -0.013) | <0.001 |
| UK | 1,163 | 0.855 | 0.282 | 1,163 | 0.812 | 0.309 | -0.043 | (-0.068, -0.018) | 0.001 |
| US | 1,146 | 0.800 | 0.323 | 1,146 | 0.725 | 0.377 | -0.074 | (-0.108, -0.041) | <0.001 |
| Uganda | 1,038 | 0.733 | 0.415 | 1,038 | 0.573 | 0.482 | -0.159 | (-0.198, -0.121) | <0.001 |
| *Overall* | 15,480 | 0.857 | 0.286 | 15,480 | 0.804 | 0.324 | -0.053 | (-0.062, -0.043) | <0.001 |

N=sample size; Mean=weighted mean; SD=weighted standard deviation; CI=confidence interval.
