# Supplementary material for: The COVID-19 pandemic and health-related quality of life across 13 high- and low-middle-income countries: A cross-sectional analysis
Source: PLoS Med. 2023 Apr 11;20(4):e1004146. doi: 10.1371/journal.pmed.1004146 (PMC10089360; doi:10.1371/journal.pmed.1004146)
Supplement: S17 Table — (DOCX) [file pmed.1004146.s017.docx]

**S17 Table. Mean difference in EQ-5D-5L index (utility) pre-COVID-19 and at time of survey,**

**India value set – Male only**

| **Country** | **EQ-5D index pre-COVID-19** | | | **EQ-5D index at survey** | | | **EQ-5D index at survey –**  **EQ-5D index pre-pandemic** | | |
| --- | --- | --- | --- | --- | --- | --- | --- | --- | --- |
|  | **N** | **Mean** | **SD** | **N** | **Mean** | **SD** | **Mean difference** | **95% CI** | **p-value** |
| Australia | 642 | 0.812 | 0.288 | 642 | 0.755 | 0.334 | -0.057 | (-0.093, -0.021) | 0.002 |
| Brazil | 706 | 0.869 | 0.270 | 706 | 0.844 | 0.285 | -0.024 | (-0.063, 0.014) | 0.215 |
| Canada | 617 | 0.862 | 0.250 | 617 | 0.799 | 0.309 | -0.063 | (-0.094, -0.032) | <0.001 |
| Chile | 436 | 0.907 | 0.254 | 436 | 0.788 | 0.311 | -0.119 | (-0.275, 0.037) | 0.135 |
| China | 683 | 0.901 | 0.214 | 683 | 0.921 | 0.148 | 0.020 | (-0.021, 0.061) | 0.334 |
| Colombia | 520 | 0.874 | 0.304 | 520 | 0.889 | 0.249 | 0.014 | (-0.045, 0.074) | 0.637 |
| France | 634 | 0.900 | 0.232 | 634 | 0.881 | 0.232 | -0.019 | (-0.046, 0.008) | 0.164 |
| India | 720 | 0.733 | 0.404 | 720 | 0.630 | 0.424 | -0.103 | (-0.145, -0.060) | <0.001 |
| Italy | 488 | 0.913 | 0.214 | 488 | 0.877 | 0.261 | -0.035 | (-0.067, -0.003) | 0.030 |
| Spain | 560 | 0.952 | 0.153 | 560 | 0.929 | 0.177 | -0.023 | (-0.042, -0.003) | 0.022 |
| UK | 625 | 0.854 | 0.289 | 625 | 0.826 | 0.307 | -0.028 | (-0.063, 0.006) | 0.108 |
| US | 580 | 0.768 | 0.355 | 580 | 0.700 | 0.402 | -0.067 | (-0.120, -0.015) | 0.011 |
| Uganda | 762 | 0.739 | 0.400 | 762 | 0.575 | 0.480 | -0.164 | (-0.208, -0.120) | <0.001 |
| *Overall* | 7,973 | 0.847 | 0.300 | 7,973 | 0.795 | 0.338 | -0.052 | (-0.067, -0.038) | <0.001 |

N=sample size; Mean=weighted mean; SD=weighted standard deviation; CI=confidence interval.
