# Supplementary material for: The COVID-19 pandemic and health-related quality of life across 13 high- and low-middle-income countries: A cross-sectional analysis
Source: PLoS Med. 2023 Apr 11;20(4):e1004146. doi: 10.1371/journal.pmed.1004146 (PMC10089360; doi:10.1371/journal.pmed.1004146)
Supplement: S18 Table — (DOCX) [file pmed.1004146.s018.docx]

**S18 Table. Mean difference in EQ-5D-5L index (utility) pre-COVID-19 and at time of survey,**

**India value set – Female and Other only**

| **Country** | **EQ-5D index pre-COVID-19** | | | **EQ-5D index at survey** | | | **EQ-5D index at survey –**  **EQ-5D index pre-pandemic** | | |
| --- | --- | --- | --- | --- | --- | --- | --- | --- | --- |
|  | **N** | **Mean** | **SD** | **N** | **Mean** | **SD** | **Mean difference** | **95% CI** | **p-value** |
| Australia | 716 | 0.838 | 0.272 | 716 | 0.798 | 0.310 | -0.040 | (-0.075, -0.005) | 0.023 |
| Brazil | 715 | 0.884 | 0.226 | 715 | 0.824 | 0.272 | -0.060 | (-0.093, -0.027) | <0.001 |
| Canada | 531 | 0.872 | 0.232 | 531 | 0.800 | 0.291 | -0.071 | (-0.103, -0.040) | <0.001 |
| Chile | 684 | 0.908 | 0.217 | 684 | 0.852 | 0.230 | -0.056 | (-0.091, -0.021) | 0.002 |
| China | 608 | 0.914 | 0.172 | 608 | 0.901 | 0.236 | -0.013 | (-0.065, 0.039) | 0.619 |
| Colombia | 711 | 0.906 | 0.238 | 711 | 0.873 | 0.247 | -0.032 | (-0.066, 0.001) | 0.06 |
| France | 508 | 0.878 | 0.272 | 508 | 0.847 | 0.290 | -0.032 | (-0.070, 0.006) | 0.103 |
| India | 470 | 0.708 | 0.413 | 470 | 0.619 | 0.440 | -0.089 | (-0.143, -0.034) | 0.001 |
| Italy | 592 | 0.908 | 0.184 | 592 | 0.872 | 0.229 | -0.036 | (-0.062, -0.011) | 0.005 |
| Spain | 592 | 0.936 | 0.186 | 592 | 0.904 | 0.193 | -0.032 | (-0.054, -0.011) | 0.003 |
| UK | 538 | 0.855 | 0.276 | 538 | 0.798 | 0.311 | -0.058 | (-0.093, -0.022) | 0.001 |
| US | 566 | 0.830 | 0.286 | 566 | 0.749 | 0.350 | -0.081 | (-0.125, -0.037) | <0.001 |
| Uganda | 276 | 0.714 | 0.454 | 276 | 0.568 | 0.486 | -0.146 | (-0.224, -0.067) | <0.001 |
| *Overall* | 7,507 | 0.867 | 0.269 | 7,507 | 0.814 | 0.308 | -0.053 | (-0.064, -0.042) | <0.001 |

N=sample size; Mean=weighted mean; SD=weighted standard deviation; CI=confidence interval.
