# Supplementary material for: The COVID-19 pandemic and health-related quality of life across 13 high- and low-middle-income countries: A cross-sectional analysis
Source: PLoS Med. 2023 Apr 11;20(4):e1004146. doi: 10.1371/journal.pmed.1004146 (PMC10089360; doi:10.1371/journal.pmed.1004146)
Supplement: S19 Table — (DOCX) [file pmed.1004146.s019.docx]

**S9 Table. National mean difference in EQ-5D-5D-5L index (utility) by age, UK tariff**

**Australia**

| Age | Utility pre-COVID-19 | | | Utility at survey | | | Utility difference | | | Population^*^ | QALY change |
| --- | --- | --- | --- | --- | --- | --- | --- | --- | --- | --- | --- |
|  | N | Mean | SD | N | Mean | SD | Mean | 95% CI | p-value |  |  |
| 18-24 years | 142 | 0.81 | 0.232 | 142 | 0.718 | 0.293 | -0.092 | (-0.153, -0.031) | 0.003 | 2,168,461 | -199,498 |
| 25-34 years | 305 | 0.766 | 0.283 | 305 | 0.715 | 0.312 | -0.051 | (-0.102, -0.000) | 0.05 | 3,549,631 | -181,031 |
| 35-44 years | 276 | 0.752 | 0.259 | 276 | 0.667 | 0.305 | -0.085 | (-0.136, -0.035) | 0.001 | 3,309,496 | -281,307 |
| 45-54 years | 188 | 0.73 | 0.302 | 188 | 0.66 | 0.346 | -0.07 | (-0.146, 0.005) | 0.066 | 3,184,780 | -222,935 |
| 55-64 years | 224 | 0.781 | 0.244 | 224 | 0.766 | 0.247 | -0.015 | (-0.067, 0.036) | 0.564 | 2,897,947 | -43,469 |
| 65+ years | 223 | 0.794 | 0.23 | 223 | 0.769 | 0.235 | -0.025 | (-0.073, 0.024) | 0.317 | 4,016,956 | -100,424 |
|  |  |  |  |  |  |  |  |  |  | **19,127,271** | **-1,028,664** |

**Brazil**

| Age | Utility pre-COVID-19 | | | Utility at survey | | | Utility difference | | | Population^*^ | QALY change |
| --- | --- | --- | --- | --- | --- | --- | --- | --- | --- | --- | --- |
|  | N | Mean | SD | N | Mean | SD | Mean | 95% CI | p-value |  |  |
| 18-24 years | 178 | 0.848 | 0.209 | 178 | 0.758 | 0.277 | -0.09 | (-0.151, -0.029) | 0.004 | 24,189,074 | -2,177,017 |
| 25-34 years | 364 | 0.803 | 0.262 | 364 | 0.723 | 0.304 | -0.08 | (-0.135, -0.024) | 0.005 | 34,675,372 | -2,774,030 |
| 35-44 years | 288 | 0.878 | 0.172 | 288 | 0.801 | 0.235 | -0.078 | (-0.120, -0.035) | <0.001 | 33,228,920 | -2,591,856 |
| 45-54 years | 246 | 0.84 | 0.211 | 246 | 0.784 | 0.279 | -0.056 | (-0.113, 0.001) | 0.055 | 26,339,990 | -1,475,039 |
| 55-64 years | 255 | 0.808 | 0.291 | 255 | 0.791 | 0.258 | -0.017 | (-0.076, 0.043) | 0.582 | 20,472,174 | -348,027 |
| 65+ years | 90 | 0.822 | 0.199 | 90 | 0.786 | 0.203 | -0.036 | (-0.111, 0.040) | 0.354 | 20,281,930 | -730,149 |
|  |  |  |  |  |  |  |  |  |  | **159,187,460** | **-10,096,118** |

**Canada**

| Age | Utility pre-COVID-19 | | | Utility at survey | | | Utility difference | | | Population^*^ | QALY change |
| --- | --- | --- | --- | --- | --- | --- | --- | --- | --- | --- | --- |
|  | N | Mean | SD | N | Mean | SD | Mean | 95% CI | p-value |  |  |
| 18-24 years | 142 | 0.802 | 0.229 | 142 | 0.657 | 0.291 | -0.146 | (-0.206, -0.085) | <0.001 | 3,018,081 | -440,640 |
| 25-34 years | 217 | 0.835 | 0.216 | 217 | 0.711 | 0.287 | -0.123 | (-0.171, -0.076) | <0.001 | 4,809,186 | -591,530 |
| 35-44 years | 200 | 0.82 | 0.238 | 200 | 0.747 | 0.292 | -0.073 | (-0.125, -0.021) | 0.006 | 4,786,308 | -349,400 |
| 45-54 years | 175 | 0.809 | 0.218 | 175 | 0.752 | 0.264 | -0.057 | (-0.107, -0.006) | 0.027 | 4,820,196 | -274,751 |
| 55-64 years | 191 | 0.784 | 0.278 | 191 | 0.716 | 0.313 | -0.068 | (-0.127, -0.009) | 0.024 | 5,194,646 | -353,236 |
| 65+ years | 223 | 0.82 | 0.203 | 223 | 0.782 | 0.212 | -0.038 | (-0.076, 0.000) | 0.052 | 6,580,761 | -250,069 |
|  |  |  |  |  |  |  |  |  |  | **29,209,178** | **-2,259,626** |

**Chile**

| Age | Utility pre-COVID-19 | | | Utility at survey | | | Utility difference | | | Population^*^ | QALY change |
| --- | --- | --- | --- | --- | --- | --- | --- | --- | --- | --- | --- |
|  | N | Mean | SD | N | Mean | SD | Mean | 95% CI | p-value |  |  |
| 18-24 years | 305 | 0.887 | 0.152 | 305 | 0.735 | 0.233 | -0.151 | (-0.218, -0.085) | <0.001 | 1,876,473 | -283,347 |
| 25-34 years | 279 | 0.894 | 0.187 | 279 | 0.79 | 0.223 | -0.104 | (-0.168, -0.040) | 0.001 | 2,918,376 | -303,511 |
| 35-44 years | 157 | 0.81 | 0.249 | 157 | 0.783 | 0.211 | -0.028 | (-0.091, 0.036) | 0.391 | 2,462,017 | -68,936 |
| 45-54 years | 222 | 0.825 | 0.276 | 222 | 0.773 | 0.279 | -0.052 | (-0.216, 0.113) | 0.536 | 2,397,264 | -124,658 |
| 55-64 years | 105 | 0.843 | 0.267 | 105 | 0.808 | 0.236 | -0.035 | (-0.112, 0.042) | 0.373 | 2,042,015 | -71,471 |
| 65+ years | 52 | 0.878 | 0.099 | 52 | 0.521 | 0.412 | -0.356 | (-0.719, 0.007) | 0.054 | 2,148,739 | -764,951 |
|  |  |  |  |  |  |  |  |  |  | **13,844,884** | **-1,616,874** |

**Colombia**

| Age | Utility pre-COVID-19 | | | Utility at survey | | | Utility difference | | | Population^*^ | QALY change |
| --- | --- | --- | --- | --- | --- | --- | --- | --- | --- | --- | --- |
|  | N | Mean | SD | N | Mean | SD | Mean | 95% CI | p-value |  |  |
| 18-24 years | 165 | 0.894 | 0.176 | 165 | 0.804 | 0.223 | -0.09 | (-0.146, -0.033) | 0.002 | 5,740,524 | -516,647 |
| 25-34 years | 321 | 0.878 | 0.211 | 321 | 0.842 | 0.219 | -0.036 | (-0.079, 0.008) | 0.11 | 7,561,311 | -272,207 |
| 35-44 years | 330 | 0.867 | 0.268 | 330 | 0.834 | 0.243 | -0.033 | (-0.090, 0.024) | 0.254 | 6,440,118 | -212,524 |
| 45-54 years | 252 | 0.841 | 0.252 | 252 | 0.838 | 0.245 | -0.004 | (-0.065, 0.058) | 0.91 | 5,550,212 | -22,201 |
| 55-64 years | 125 | 0.871 | 0.232 | 125 | 0.859 | 0.222 | -0.012 | (-0.077, 0.052) | 0.705 | 4,475,682 | -53,708 |
| 65+ years | 38 | 0.768 | 0.33 | 38 | 0.769 | 0.298 | 0.002 | (-0.176, 0.179) | 0.986 | 4,615,469 | 9,231 |
|  |  |  |  |  |  |  |  |  |  | **34,383,316** | **-1,068,056** |

**France**

| Age | Utility pre-COVID-19 | | | Utility at survey | | | Utility difference | | | Population^*^ | QALY change |
| --- | --- | --- | --- | --- | --- | --- | --- | --- | --- | --- | --- |
|  | N | Mean | SD | N | Mean | SD | Mean | 95% CI | p-value |  |  |
| 18-24 years | 91 | 0.841 | 0.219 | 91 | 0.748 | 0.279 | -0.093 | (-0.166, -0.019) | 0.013 | 5,481,459 | -509,776 |
| 25-34 years | 165 | 0.867 | 0.21 | 165 | 0.817 | 0.235 | -0.049 | (-0.101, 0.003) | 0.064 | 7,776,086 | -381,028 |
| 35-44 years | 156 | 0.88 | 0.196 | 156 | 0.836 | 0.212 | -0.043 | (-0.089, 0.002) | 0.062 | 8,265,605 | -355,421 |
| 45-54 years | 205 | 0.853 | 0.234 | 205 | 0.81 | 0.252 | -0.043 | (-0.095, 0.010) | 0.11 | 8,836,577 | -379,973 |
| 55-64 years | 277 | 0.831 | 0.258 | 277 | 0.804 | 0.234 | -0.027 | (-0.075, 0.021) | 0.27 | 8,264,917 | -223,153 |
| 65+ years | 248 | 0.817 | 0.251 | 248 | 0.774 | 0.275 | -0.043 | (-0.095, 0.009) | 0.105 | 13,303,244 | -572,039 |
|  |  |  |  |  |  |  |  |  |  | **51,927,888** | **-2,421,390** |

**India**

| Age | Utility pre-COVID-19 | | | Utility at survey | | | Utility difference | | | Population^*^ | QALY change |
| --- | --- | --- | --- | --- | --- | --- | --- | --- | --- | --- | --- |
|  | N | Mean | SD | N | Mean | SD | Mean | 95% CI | p-value |  |  |
| 18-24 years | 282 | 0.718 | 0.337 | 282 | 0.622 | 0.371 | -0.096 | (-0.154, -0.038) | 0.001 | 182,990,512 | -17,567,089 |
| 25-34 years | 508 | 0.697 | 0.357 | 508 | 0.581 | 0.347 | -0.116 | (-0.159, -0.073) | <0.001 | 230,764,784 | -26,768,715 |
| 35-44 years | 215 | 0.687 | 0.361 | 215 | 0.594 | 0.359 | -0.093 | (-0.161, -0.025) | 0.007 | 188,994,832 | -17,576,519 |
| 45-54 years | 99 | 0.723 | 0.367 | 99 | 0.598 | 0.391 | -0.125 | (-0.230, -0.020) | 0.019 | 139,650,192 | -17,456,274 |
| 55-64 years | 62 | 0.795 | 0.304 | 62 | 0.662 | 0.297 | -0.133 | (-0.238, -0.028) | 0.013 | 98,145,792 | -13,053,390 |
| 65+ years | 24 | 0.714 | 0.407 | 24 | 0.62 | 0.445 | -0.094 | (-0.330, 0.142) | 0.437 | 93,164,416 | -8,757,455 |
|  |  |  |  |  |  |  |  |  |  | **933,710,528** | **-101,179,443** |

**Italy**

| Age | Utility pre-COVID-19 | | | Utility at survey | | | Utility difference | | | Population^*^ | QALY change |
| --- | --- | --- | --- | --- | --- | --- | --- | --- | --- | --- | --- |
|  | N | Mean | SD | N | Mean | SD | Mean | 95% CI | p-value |  |  |
| 18-24 years | 89 | 0.781 | 0.292 | 89 | 0.709 | 0.327 | -0.072 | (-0.167, 0.022) | 0.134 | 4,127,764 | -297,199 |
| 25-34 years | 159 | 0.823 | 0.245 | 159 | 0.763 | 0.294 | -0.06 | (-0.122, 0.002) | 0.057 | 6,559,388 | -393,563 |
| 35-44 years | 221 | 0.885 | 0.147 | 221 | 0.821 | 0.208 | -0.064 | (-0.098, -0.029) | <0.001 | 8,121,335 | -519,765 |
| 45-54 years | 228 | 0.868 | 0.195 | 228 | 0.823 | 0.223 | -0.045 | (-0.085, -0.005) | 0.028 | 9,779,625 | -440,083 |
| 55-64 years | 241 | 0.869 | 0.17 | 241 | 0.817 | 0.235 | -0.052 | (-0.093, -0.011) | 0.013 | 8,274,090 | -430,253 |
| 65+ years | 142 | 0.868 | 0.182 | 142 | 0.841 | 0.174 | -0.026 | (-0.070, 0.017) | 0.237 | 13,784,435 | -358,395 |
|  |  |  |  |  |  |  |  |  |  | **50,646,637** | **-2,439,259** |

**Spain**

| Age | Utility pre-COVID-19 | | | Utility at survey | | | Utility difference | | | Population^*^ | QALY change |
| --- | --- | --- | --- | --- | --- | --- | --- | --- | --- | --- | --- |
|  | N | Mean | SD | N | Mean | SD | Mean | 95% CI | p-value |  |  |
| 18-24 years | 50 | 0.913 | 0.217 | 50 | 0.85 | 0.211 | -0.063 | (-0.146, 0.020) | 0.139 | 3,091,684 | -194,776 |
| 25-34 years | 226 | 0.917 | 0.13 | 226 | 0.844 | 0.191 | -0.073 | (-0.103, -0.043) | <0.001 | 5,137,782 | -375,058 |
| 35-44 years | 209 | 0.911 | 0.16 | 209 | 0.857 | 0.186 | -0.054 | (-0.088, -0.021) | 0.001 | 7,309,586 | -394,718 |
| 45-54 years | 253 | 0.9 | 0.178 | 253 | 0.84 | 0.204 | -0.06 | (-0.094, -0.027) | <0.001 | 7,342,949 | -440,577 |
| 55-64 years | 274 | 0.883 | 0.206 | 274 | 0.86 | 0.183 | -0.023 | (-0.056, 0.009) | 0.16 | 5,993,525 | -137,851 |
| 65+ years | 140 | 0.899 | 0.173 | 140 | 0.86 | 0.194 | -0.039 | (-0.082, 0.004) | 0.072 | 8,999,257 | -350,971 |
|  |  |  |  |  |  |  |  |  |  | **37,874,783** | **-1,893,951** |

**UK**

| Age | Utility pre-COVID-19 | | | Utility at survey | | | Utility difference | | | Population^*^ | QALY change |
| --- | --- | --- | --- | --- | --- | --- | --- | --- | --- | --- | --- |
|  | N | Mean | SD | N | Mean | SD | Mean | 95% CI | p-value |  |  |
| 18-24 years | 57 | 0.792 | 0.277 | 57 | 0.695 | 0.295 | -0.097 | (-0.200, 0.007) | 0.068 | 5,604,396 | -543,626 |
| 25-34 years | 171 | 0.82 | 0.27 | 171 | 0.767 | 0.277 | -0.053 | (-0.110, 0.005) | 0.074 | 9,457,326 | -501,238 |
| 35-44 years | 221 | 0.784 | 0.289 | 221 | 0.736 | 0.301 | -0.048 | (-0.105, 0.009) | 0.099 | 8,716,166 | -418,376 |
| 45-54 years | 200 | 0.799 | 0.249 | 200 | 0.741 | 0.282 | -0.057 | (-0.111, -0.004) | 0.037 | 9,189,565 | -523,805 |
| 55-64 years | 260 | 0.794 | 0.288 | 260 | 0.741 | 0.307 | -0.053 | (-0.105, -0.001) | 0.044 | 8,079,824 | -428,231 |
| 65+ years | 254 | 0.826 | 0.217 | 254 | 0.789 | 0.224 | -0.037 | (-0.076, 0.002) | 0.062 | 12,123,632 | -448,574 |
|  |  |  |  |  |  |  |  |  |  | **53,170,909** | **-2,863,851** |

**US**

| Age | Utility pre-COVID-19 | | | Utility at survey | | | Utility difference | | | Population^*^ | QALY change |
| --- | --- | --- | --- | --- | --- | --- | --- | --- | --- | --- | --- |
|  | N | Mean | SD | N | Mean | SD | Mean | 95% CI | p-value |  |  |
| 18-24 years | 67 | 0.693 | 0.362 | 67 | 0.651 | 0.347 | -0.042 | (-0.170, 0.086) | 0.519 | 30,367,254 | -1,275,425 |
| 25-34 years | 220 | 0.72 | 0.293 | 220 | 0.615 | 0.339 | -0.105 | (-0.171, -0.039) | 0.002 | 45,025,396 | -4,727,667 |
| 35-44 years | 272 | 0.727 | 0.306 | 272 | 0.606 | 0.356 | -0.121 | (-0.184, -0.058) | <0.001 | 41,647,780 | -5,039,381 |
| 45-54 years | 165 | 0.802 | 0.255 | 165 | 0.722 | 0.318 | -0.08 | (-0.149, -0.010) | 0.024 | 41,474,136 | -3,317,931 |
| 55-64 years | 213 | 0.758 | 0.281 | 213 | 0.703 | 0.322 | -0.055 | (-0.124, 0.013) | 0.111 | 42,938,392 | -2,361,612 |
| 65+ years | 209 | 0.81 | 0.231 | 209 | 0.769 | 0.257 | -0.04 | (-0.101, 0.021) | 0.195 | 53,495,832 | -2,139,833 |
|  |  |  |  |  |  |  |  |  |  | **254,948,790** | **-18,861,848** |

**Uganda**

| Age | Utility pre-COVID-19 | | | Utility at survey | | | Utility difference | | | Population^*^ | QALY change |
| --- | --- | --- | --- | --- | --- | --- | --- | --- | --- | --- | --- |
|  | N | Mean | SD | N | Mean | SD | Mean | 95% CI | p-value |  |  |
| 18-24 years | 269 | 0.719 | 0.365 | 269 | 0.573 | 0.39 | -0.146 | (-0.209, -0.082) | <0.001 | 5,705,615 | -833,020 |
| 25-34 years | 587 | 0.727 | 0.352 | 587 | 0.563 | 0.407 | -0.164 | (-0.208, -0.121) | <0.001 | 5,531,647 | -907,190 |
| 35-44 years | 145 | 0.761 | 0.338 | 145 | 0.615 | 0.397 | -0.146 | (-0.230, -0.061) | 0.001 | 3,415,678 | -498,689 |
| 45-54 years | 27 | 0.731 | 0.474 | 27 | 0.427 | 0.529 | -0.304 | (-0.567, -0.042) | 0.023 | 2,143,713 | -651,689 |
| 55-64 years | 8 | 0.688 | 0.459 | 8 | 0.566 | 0.369 | -0.122 | (-0.504, 0.259) | 0.529 | 1,250,801 | -152,598 |
| 65+ years | 2 | 0.824 | 0.071 | 2 | 0.839 | 0.099 | 0.016 | (-0.104, 0.135) | 0.796 | 972,780 | 15,564 |
|  |  |  |  |  |  |  |  |  |  | **19,020,234** | **-3,027,621** |
