# Supplementary material for: The COVID-19 pandemic and health-related quality of life across 13 high- and low-middle-income countries: A cross-sectional analysis
Source: PLoS Med. 2023 Apr 11;20(4):e1004146. doi: 10.1371/journal.pmed.1004146 (PMC10089360; doi:10.1371/journal.pmed.1004146)
Supplement: S2 Fig — (DOCX) [file pmed.1004146.s021.docx]

**S2 Fig.** **Paretian Classification of Health Change (PCHC) by World Bank income classification group (ICG)**
