# Supplementary material for: The COVID-19 pandemic and health-related quality of life across 13 high- and low-middle-income countries: A cross-sectional analysis
Source: PLoS Med. 2023 Apr 11;20(4):e1004146. doi: 10.1371/journal.pmed.1004146 (PMC10089360; doi:10.1371/journal.pmed.1004146)
Supplement: S10 Fig — (DOCX) [file pmed.1004146.s029.docx]

**S10 Fig. Worsened anxiety/depression by age and gender for each continent**

| Panel a – Africa | Panel b – Asia | Panel c – Europe |
| --- | --- | --- |
| Panel d – North America | Panel e – Oceania | Panel d – South America |
