# Supplementary material for: The COVID-19 pandemic and health-related quality of life across 13 high- and low-middle-income countries: A cross-sectional analysis
Source: PLoS Med. 2023 Apr 11;20(4):e1004146. doi: 10.1371/journal.pmed.1004146 (PMC10089360; doi:10.1371/journal.pmed.1004146)
Supplement: S11 Fig — (DOCX) [file pmed.1004146.s030.docx]

**S11 Fig. Worsened health by EQ-5D domains but anxiety/depression – stratified for by age and gender**

| Panel a – Mobility | Panel b – Self-care |
| --- | --- |
| Panel c – Usual activities | Panel d – Pain/discomfort |
