# Supplementary material for: The COVID-19 pandemic and health-related quality of life across 13 high- and low-middle-income countries: A cross-sectional analysis
Source: PLoS Med. 2023 Apr 11;20(4):e1004146. doi: 10.1371/journal.pmed.1004146 (PMC10089360; doi:10.1371/journal.pmed.1004146)
Supplement: Attachment — Submitted filename: Comments on PMEDICINE-D-22-01975_R1.docx [file pmed.1004146.s032.docx]

Comments on the paper “The impact of the COVID-19 pandemic on health-related quality of life: a cross-sectional survey of 13 high and low-middle income countries” (Ref No: PMEDICINE-D-22-01975R1)

This is an interesting and informative paper. I have following comments on the paper:

1. My first suggestion is- please cite all the references within sentences. For example, you have cited references either outside full-stops (.) or outside commas (,) which do not look right.
2. In the Methods section, under ‘Procedures’, you have mentioned- “QALYs lost at population levels were estimated from the UK-valued EQ-5D-5L and external data on population sizes.” Could you please give justifications for this as the value sets may differ greatly by study country? This may affect validity of your analysis results.
3. In the Methods section, under ‘Statistical Analyses’, in the last paragraph you have mentioned- “The resulting country level QALY loss due to COVID-19 pandemic-related morbidity was then expressed as a ratio of country-level QALY loss due to COVID-19 premature mortality, estimated by multiplying confirmed country-level COVID-19 deaths by current estimates of QALYs lost per death.(22-24)”. Method explained in this sentence is not very clear to readers. Please expand how did you calculate QALYs lost per death for every country, to make it clear to readers?
4. In addition to above comment, in Table 3, you have used same lower and upper QALYs lost per death values for every country. How do you justify this as we know this may differ in every country?
5. In the Results section (page 10), you have reported – “For all other health domains, younger groups remained most impacted, but deterioration in health was, on average, greater for males (*Figure S11)*.” Could you please explain the reasons why deterioration in health was greater for males than females?
6. In the Results section, under “HRQoL changes*”* (page 11), you have mentioned- “Only China showed non-statistically significant changes”. Why? Do you know any reasons why China showed non-statistically significant changes?
7. In Table 1, could you please explain why female and other gender categorised in one group? Also, what types of gender come under ‘other’, any examples? Please define ‘other’ gender in the body of the paper too so that readers are clear about it.
8. In Table 1, average age of respondents from Africa reported as 29.11 yrs but other continent over 42 yrs. I doubt the sample taken from Africa may be representative of the study population (may be due to that reason, income loss from Africa is very high (88.73%) compared to other countries, as shown in Table 1). What is your opinion about the representativeness of sample from Africa? How do you convince the sample from Africa is representative of the study population?
9. Again in Table 1, in co-morbidities categories, one category is- 'Allergies', which is too vague to understand by readers. Can you please give some examples in a bracket what are covered under ‘Allergies’ so that readers are clearer about this?

Thank you.
