# Supplementary material for: The COVID-19 pandemic and health-related quality of life across 13 high- and low-middle-income countries: A cross-sectional analysis
Source: PLoS Med. 2023 Apr 11;20(4):e1004146. doi: 10.1371/journal.pmed.1004146 (PMC10089360; doi:10.1371/journal.pmed.1004146)
Supplement: Attachment — Submitted filename: Response to Reviewers_updated.docx [file pmed.1004146.s033.docx]

**Manuscript PMEDICINE-D-22-01975R1: Responses to Editors’ and Reviewers’ comments**

**NOTE:** Editors’ and Reviewers’ comments are shown in **bold**, with our responses shown below each. We also report in our responses relevant edited extracts from the revised manuscript in *Italics*.

**REQUESTS FROM THE EDITORS:**

We thank the editors for their suggestions aimed at improving our manuscript.

**Please include continuous line numbering throughout your manuscript to facilitate review.**

Line numbers have been added throughout the manuscript as requested.

**Please update your title to ‘The COVID-19 pandemic and health-related quality of life across 13 high and low-middle income countries: a cross-sectional analysis’, or similar.**

The title has been updated to ‘The COVID-19 pandemic and health-related quality of life across 13 high and low-middle income countries: a cross-sectional analysis’ as requested.

**PLOS Medicine requires that the de-identified data underlying the specific results in a published article be made available, without restrictions on access, in a public repository or as Supporting Information at the time of article publication, provided it is legal and ethical to do so. Please see the policy at** [**http://journals.plos.org/plosmedicine/s/data-availability**](http://journals.plos.org/plosmedicine/s/data-availability) **and FAQs at** [**http://journals.plos.org/plosmedicine/s/data-availability#loc-faqs-for-data-policy**](http://journals.plos.org/plosmedicine/s/data-availability#loc-faqs-for-data-policy)

We acknowledge the importance of sharing data and we have indicated where data are made publicly available:

*<< All 42 files are available from the Dataverse Repository database (accession number(s) <https://doi.org/10.7910/DVN/PMV0TG>) https://dataverse.harvard.edu/dataset.xhtml?persistentId=doi:10.7910/DVN/PMV0TG>>.*

**Your study is observational and therefore causality cannot be inferred. Please remove language that implies causality, such as ‘impact’ or ‘affect’. Refer to associations instead.**

Language implying causality in our results has been adjusted throughout the manuscript, referring to associations and/or other appropriate language. See for example page 2, line 31 and 36.

**Abstract Background:**

**Provide the context of why the study is important. The final sentence should clearly state the study question.**

Additional context has been provided on page 2, line 29-30, outlining the importance of the study:

*<< It is also important to consider HRQoL to better understand the potential multi-faceted implications of the COVID-19 pandemic in various international contexts>>.*

The final sentence of the abstract Background (page 2, lines 30-32) also clearly states the aim of the study:

*<< This study aimed to assess the association between the COVID-19 pandemic and changes in HRQoL in 13 diverse countries >>*.

**Abstract Methods and Findings:**

*** Please ensure that all numbers presented in the abstract are present and identical to numbers presented in the main manuscript text.**

We can confirm that all of the numbers presented in the abstract are present and identical to the numbers presented in the main manuscript text.

*** Please include the study design (cross-sectional) and population and setting.**

The study design and population setting have been added to the abstract on page 2, lines 33-35:

*<<Adults (18+ years) were surveyed online (24 November - 17 December 2020)* ***in 13 countries spanning six continents****.* ***Our cross-sectional study*** *used descriptive and regression-based analyses…>>.*

*** In the last sentence of the Abstract Methods and Findings section, please describe the main limitation(s) of the study's methodology.**

The main limitation of the study has been added to the end of the methods and findings section of the abstract, on page 2, lines 48-50:

*<< A limitation of the study is that subjects were asked to complete the pre-pandemic health questionnaire retrospectively, meaning responses may be subject to recall bias>>.*

**Abstract Conclusions:**

*** Please address the study implications without overreaching what can be concluded from the data; the phrase "In this study, we observed ..." may be useful.**

Additional text on the implications of the study has been added to the abstract conclusions, on page 3, line 51-53:

*<< In this study, we observed that the COVID-19 pandemic was associated with a reduction in perceived HRQoL globally, especially with respect to the anxiety/depression health domain and amongst younger people>>.*

**At this stage, we ask that you include a short, non-technical Author Summary of your research to make findings accessible to a wide audience that includes both scientists and non-scientists. The Author Summary should immediately follow the Abstract in your revised manuscript. This text is subject to editorial change and should be distinct from the scientific abstract. Please see our author guidelines for more information:** [**https://journals.plos.org/plosmedicine/s/revising-your-manuscript#loc-author-summary**](https://journals.plos.org/plosmedicine/s/revising-your-manuscript#loc-author-summary)

**Why was this study done?**

- The health burden of the COVID-19 pandemic has been closely tracked in terms of confirmed cases and deaths due to the virus.
- Data on the relationship between the COVID-19 pandemic and health-related quality of life of the general population globally is still quite limited.
- Existing studies have mainly focussed on single countries and used relatively small convenience samples, making cross-country comparison difficult.
- It is important to understand similarities and differences in the association between the COVID-19 pandemic and health-related quality of life in different countries around the world, because this information can help policy-makers to find the most appropriate interventions that work in diverse settings, health conditions, and populations, to combat the ongoing COVID-19 pandemic and potential future pandemics.

**What did the researchers do and find?**

- We asked 15,536 participants from a diverse group of 13 countries (approx. 1,200 per country) to answer an online survey in which we asked questions about socio-demographic characteristics, health, and perceived health-related quality of life prior to and during the pandemic.
- We found that the COVID-19 pandemic was associated with significantly worse health-related quality of life for more than one third of respondents, with anxiety/depression being the aspect of health that worsened the most, especially for younger people (<35 years) and females.
- Overall, our results suggest that there was an 8% reduction in perceived health-related quality of life, and the health burden, measured in quality-adjusted life years, associated with morbidity was considerably higher than that due to premature, COVID-19-related, mortality.

**What do these findings mean?**

- The health burden of the COVID-19 pandemic may be substantially underestimated if we consider mortality alone.
- Health-related quality of life measures are important to fully capture the considerable health burden of the COVID-19 pandemic and its associated containment policies.
- Our results provide benchmark evidence for countries at different stages in the pandemic and can help inform public health measures and economic policies in the event of other health shocks in the future.

**Please place citations within square brackets and before punctuation.**

Citations have been placed within square brackets and before punctuation throughout the revised manuscript.

**Please specify whether informed consent was written or oral.**

Informed consent was written, which has been clarified on pages 5/6, lines 125-126:

*<<All participants provided informed* ***written*** *consent at the beginning of the survey>>.*

**Please ensure that the study is reported according to the STROBE guideline, and include the completed STROBE checklist as Supporting Information. Please add the following statement, or similar, to the Methods: "This study is reported as per the Strengthening the Reporting of Observational Studies in Epidemiology (STROBE) guideline (S1 Checklist)."**

**The STROBE guideline can be found here:** [**http://www.equator-network.org/reporting-guidelines/strobe/**](http://www.equator-network.org/reporting-guidelines/strobe/)

**When completing the checklist, please use section and paragraph numbers, rather than page numbers.**

The STROBE checklist has been created and added to the supplementary information (S1 Table), and a statement has been added to the methods on page 6, lines 125-127:

*<< This study is reported as per the Strengthening the Reporting of Observational Studies in Epidemiology (STROBE) guideline (Table S19)>>.*

**Please provide the rationale behind grouping gender as ‘Male’ and ‘Female & Other’.**

We thank the Editors for giving us the opportunity to clarify this important point. Out of the 15,536 respondents, only 34 (0.2%) reported their gender as ‘Other’, which was a category approved by the University of Oxford Ethics Committee to include the gender of all individuals who did not identified either as ‘Male’ or ‘Female’.

We believed it was very important to keep these respondents rather than exclude them, but low numbers and the associated lack of statistical power prohibited separate analyses for this sub-group of our sample. Exploration of data suggested that there was not a clear pattern and in fact sample sizes were too small to draw any meaningful conclusion. We have added some text to explain this and define the category ‘Other’, on page 8, line 184-188:

*<< Because of small numbers, respondents who reported their gender as ‘Other’ were combined with those who identified as ‘Female’. Exploration of data suggested that there was not a clear pattern and in fact sample sizes were too small to draw any meaningful conclusion. The category ‘Other’ included all individuals who did not identified either as ‘Male’ or ‘Female’ >>.*

**Please avoid overstating your findings in the concluding paragraph - temper statement such as ‘Our findings show’ by referring to suggestions, to acknowledge the limitations inherent to the study design.**

The opening sentence in the concluding paragraph has been tempered to acknowledge the limitations inherent to the study design - see page 19, lines 465-467:

*<<****Our findings suggest that****, when translated into QALYs, the COVID-19 pandemic-related burden* ***associated with*** *morbidity* ***may be*** *substantial compared with plausible QALY loss due to COVID-19 premature death>>.*

**The ‘Ethics Approval’, ‘Declarations of interests’ and funding information from the ‘Acknowledgements’ section can all be removed, as the relevant information is presented either in the Submission Form or the Methods.**

The ‘Ethics Approval’, ‘Declarations of interests’ and funding information from the ‘Acknowledgements’ section have all been removed.

**Ensure date of citation is presented for internet sources in the references. See** [**https://journals.plos.org/plosmedicine/s/submission-guidelines#loc-references**](https://journals.plos.org/plosmedicine/s/submission-guidelines#loc-references) **for more information.**

We have now made sure that date of citation is presented for all internet sources.

**Figure S12 – overlapping text makes some of the data labels difficult to read.**

Data labels have been removed from S12 Figure as some of them were not legible, and the relevant data can be found in S10 Table, which is presented directly below S12 Figure in the Supplementary information.

REVIEWER #1:

**Thanks for the opportunity to review your manuscript. My role is as a statistical reviewer, so my review concentrates on the study design, data, and analysis that are presented. I have put general questions first, followed by queries relevant to a specific section of the manuscript (with a page/paragraph reference).**

We thank Reviewer 1 for their constructive suggestions, which have helped us to improve the quality of our manuscript.

**This study examines how perceived health-related quality of life changed over the first year of the COVID-19 pandemic, and examines how this was associated with individual and national level characteristics. Participants were recruited online (most countries) and from household sampling of urban communities, with quota sampling used. For some countries, post-stratification weights were created with raking, based on region (within country), age, sex and education. Based on the info from the previous PNAS publication the relative size of the weights would appear to be country specific (e.g. more correction needed for relative under sampling of young age-groups in the USA compared to the other raking variables). CANDOUR is very interesting - and in this manuscript I particularly like the approach of considering 'health' to be more than just incidence of (one) disease and mortality, and the focus on LMICs.**

We thank the Reviewer for appreciating the importance of our study. Just to confirm, all participants were recruited online and responded to the survey online, as explained in our Methods section, on page 5, lines 114-115:

<< *Anonymous* ***online*** *surveys were completed by adults aged 18 years or more across the 13 participating countries,…*>>

**It looks as though the CANDOUR project and several of the publications have a pre-registered analysis plan available from the project website but I couldn't see one specific for this manuscript. Is one available to be included in the review process?**

As outlined in the methods section, page 5, lines 123-125, the overall study protocol - including also the content of this manuscript - was pre-registered, with a reference provided, i.e.:

*<<* *This study protocol was pre-registered [16], and approved by the University of Oxford Medical Sciences Interdivisional Research Ethics Committee (ID: R72328/RE001)>>.*

The reference provided is:

*Clarke P, Duch RM, Roope L, Violato M. CANDOUR Pre-Registration: COVID-19 Vaccine Preference and Opinion Survey. 2020 [cited 26/02/2022]. Available from:* [*https://osf.io/8ks6x/*](https://osf.io/8ks6x/)*.*

**It might be helpful to be specific about the period over which the perceived change in QoL occurred - the data was collected in late 2020, there might be a standard way to describe this period of time ('early COVID pandemic period'?) or maybe qualify the change was in the first year.**

Thank you for your suggestion. We feel as though November-December 2020, when the data were collected, is not particularly well captured by the term ‘early COVID pandemic period’, which we feel would better capture the initial global lockdowns in March 2020. We have decided to describe the time covered in the survey, i.e. pre-pandemic up to December 2020, as ‘within the first year of the COVID-19 pandemic’, as you also suggest. We have clarified this in the manuscript in the first paragraph of the methods section, page 5, lines 116-117:

*<<…..between 24 November 2020 and 17 December 2020,* ***i.e. within the first year of the COVID-19 pandemic*** *>>.*

**If post-stratification weights are applied to even part of the data, then the raw numerator for an indicator won't match the proportion reporting a particular indicator. I would just report the %, and preferably with a 95% CI (with your sample size the CI will be precise, nothing wrong with 'flaunting' that).**

Thank you for noting this. All relevant Tables in the main manuscript (Table 1) and in the Supplementary information (i.e. S2, S3, S4, and S5 Tables ) have been updated accordingly. Text in the manuscript has been updated, as appropriate.

**Is there any material available which describes the sampling process of the two countries where in-person surveys were completed? Was the sampling frame for these countries truly national or was it based in cities/areas of convenience? I also think that the labels for these two countries might need to be specific about the population, my own experience with survey research in India was that there are non-trivial difference differences between urban and village populations, and also between different areas of the country.**

Just to confirm, all participants were recruited online and responded to the survey online, as explained in our Methods section, page 5 lines 114-115:

<< *Anonymous* ***online*** *surveys were completed by adults aged 18 years or more across the 13 participating countries,…*>>

Material that explains in detail the sampling process within the CANDOUR study can be found in the Supplementary Information of our prior publication:

*Duch R, Roope LSJ, Violato M, Fuentes Becerra M, Robinson TS, Bonnefon JF, et al. Citizens from 13 countries share similar preferences for COVID-19 vaccine allocation priorities. Proc Natl Acad Sci U S A. 2021;118(38). doi: 10.1073/pnas.2026382118. PubMed PMID: 34526400; PubMed Central PMCID: PMC8463843*

to which we point both the Reviewer and the interested readers. We have clarified this in the revised manuscript in the methods section, page 5, lines 121 -123:

*<<* *Full technical details of the CANDOUR study sample, including sampling and weighting procedures are described at length in one of our cognate publications to which we point the interested reader [17] >>.*

We have also amended the text in the methods section (page 5, lines 117-118) and discussion section considering limitations (page 15, lines 356-359) to make clear that the samples from India and Uganda are not nationally representative. The edits read as follows:

Methods - page 5, lines 117-118:

*<<Except for India and Uganda, where samples were not nationally representative, including mainly urban settings,…>>*

Discussion - page 14, lines 355-358:

*<<While samples are generally representative on key socio-demographic/geographical factors in the included high-income countries,* ***the same cannot be claimed for low/middle-income countries, with India and Uganda, for example, being primarily sampled from urban populations***>>.

**One limitation of the study was that the change in HR-QOL is based off recall of participants rather than longitudinal measurement. The manuscript is appropriately worded (use of 'perceived deterioration'), but I think the limitations of this measurement should be put into the discussion section so that it's clear.**

The limitations of this retrospective measurement of HRQoL is discussed in detail in the discussion section (pages 14/15, lines 327-353), as well as mentioned in the abstract (page 2, lines 47-49).

Methods - pages 14/15, lines 327-353:

*<<Caution needs to be exerted when interpreting our findings. Findings may be biased by the fact that subjects were asked to complete the health questionnaire retrospectively and, therefore, responses may be subject to ‘recall bias’. Recall bias is a potentially complex phenomenon whereby, depending on context, people may over-weight salient events in the past or under-weight previous health problems with regards to their current health and demographic variables [27]. Current health may act as a lens or filter which affects how people recall the past [28]. The literature on recall accuracy using the EQ-5D is limited, and studies tend to be for specific populations and conditions [29-31]. Patients have also been found to recall their baseline HRQoL as being better than it actually was [32], which, in our study, would suggest the differences we observed within the first year of the COVID-19 pandemic were conservative. Furthermore, retrospective EQ-5D has been used previously in surveys for both COVID-19 [33, 34] and other diseases [35]. Compared with pre-pandemic norms in countries where EQ-5D-5L population norms are available, in Australia [36], Canada [37], China [38], Colombia [39], Spain [40], and the US [6], our retrospective HRQoL values are relatively low, even when the country-specific value sets were used (i.e. the US). If there is some downward bias in our baseline population average HRQoL estimates, all else equal, this may at least have the advantage of helping to mitigate any upward bias in our estimates of HRQoL fall. However, it also suggests that our sample may not be as representative as hoped, although HRQoL measures based on online responses have been found to be lower than face-to-face responses [6], and none of the aforementioned population norms are based on online responses. It is also worth noting that the EQ-5D-5L asks individuals to rate their current health status at the time of the survey [8], meaning responses are likely to be influenced by the context of the pandemic in each country at the time of the survey, conducted between 24 November 2020 and 17 December 2020. Given that this period coincided with a new wave of COVID-19 cases and deaths globally [41], respondents may have perceived their HRQoL as lower than they would have at other points during the pandemic, when new cases and deaths were lower>>.*

Abstract - page 2, lines 48-50:

*<<A limitation of the study is that subjects were asked to complete the pre-pandemic health questionnaire retrospectively, meaning responses may be subject to recall bias>>.*

**P2, Paragraph 2. In the methods it describes in-person sampling for two countries - but the description here only mentions the online surveys.**

We can confirm that no in-person sampling was undertaken for this study, and have ensured that the methods make this clear on page 5, lines 114-115:

*<< Anonymous* ***online*** *surveys were completed by adults aged 18 years or more across the 13 participating countries…>>*

**P5, Paragraph 1. What criteria did you use to decide that a sample from a country was imbalanced?**

As explained in our response to one of the Reviewer’s earlier general questions, material that explains in detail the sampling process within the CANDOUR study can be found in the Supplementary Information of our prior publication:

*Duch R, Roope LSJ, Violato M, Fuentes Becerra M, Robinson TS, Bonnefon JF, et al. Citizens from 13 countries share similar preferences for COVID-19 vaccine allocation priorities. Proc Natl Acad Sci U S A. 2021;118(38). doi: 10.1073/pnas.2026382118. PubMed PMID: 34526400; PubMed Central PMCID: PMC8463843*

to which we point both the Reviewer and interested readers. We have clarified this in the revised manuscript in the methods section, page 5, lines 121 -123:

*<<* *Full technical details of the CANDOUR study sample, including sampling and weighting procedures are described at length in one of our cognate publications to which we point the interested reader [17] >>.*

**P7, Paragraph 1. What was the rationale for a) multivariable models, and b) performing variable selection for the multivariable models? I can follow Models 1 and 2, but I was unsure about why the more complex multivariable model and model selection procedure was required. If the aim was to reduce confounding between the covariates, then a criteria of p<0.05 is probably too stringent as it's possible for important levels of confounding to occur where a covariate-outcome relationship has a p-value >0.05.  Was p>0.05 also the criteria for the backward selection procedure? For validation of the stepwise procedures, I would suggest a larger p-value and also bootstrapping the stepwise procedure ('swboot' in Stata does this). Was the stepwise proceure needed as there were too many parameters for the sample size? Based on the sample size (n>1000 for each country) is it possible to just include multivariable models with covariates of interest, or were there issues with collinearity or inflated SE?**

We thank the Reviewer for raising this point as it gave us the opportunity to revise the text to make clearer about the model selection process. Firstly, we agree with the Reviewer that not all of our models were necessary, given the explorative nature of these simple analyses. We have therefore dropped multivariable Model 4 and the associated backward selection procedure, as it did not convey any additional information compared with multivariable Model 3. The text referring to Model 4 has been deleted throughout the manuscript too.

We have, however, retained Model 3 (fully adjusted model) when exploring the association between “worsened health and participants’ sociodemographic and clinical characteristics” (Table S5), and between “worsened health and participants’ experiences of/exposure to COVID-19” (Table S6). This is because reporting the fully adjusted model with the variables of interest after having reported the raw association and the partially adjusted model, seemed the natural next step to complete our explorative analyses –and  no ‘backward model selection’ was applied there because not needed, as noted by the Reviewer.

For Model 3, the aim of the stepwise procedure was indeed to reduce confounding between the covariates. However, the Reviewer is correct that a criteria of p<0.05 for variable selection was too stringent, and in fact we have used the criteria of p<0.10 and have now identified and rectified both in the text and in the Tables/Figures, by re-running the correct model specifications M3. Those now include all covariates with p<0.10 in Model 2, namely all of the relevant variables, except for the model for Males in Table S5, where employment had p≥ 0.10.

The Reviewer can view the updated Tables S5 and S6 in the Supplementary information, and the relevant edited text in the Methods, on page 8, lines 188-192, reads as follows:

*<< For each set of predictors, we first estimated the unadjusted association with each predictor (Model 1); we then adjusted it by age and country (Model 2); and finally, predictors whose association had a* ***p-value < 0.10****, were included in the multivariable logistic regression (Model 3). Model 3 was not performed for national-level variables, as some indicators were nested within each other>>.*

**P8, Paragraph 1. Was missing-as-indicator used for analyses where participants had missing individual-level data?  Missing-as-indicator can work for missing-at-random data when there is no correlation between missingness and the values of other covariates (e.g. the treatment effect adjusted for covariates in an RCT). Was this the case for the sample in this study?**

Page 8, paragraph 1 of the original manuscript is about estimating QALYs lost to morbidity. As explained in the first paragraph of the results section (page 9, lines 217-218):

*<On average 1195 individuals per country participated in the CANDOUR study, a total of 15536 participants.* ***Of these, all completed the EQ-5D-5L descriptive system****>>.*

all of the respondents included in the final sample (i.e. those with complete age and gender data) completed the EQ-5D-5L descriptive system. Therefore, missing data was not an issue when estimating QALYs lost associated to morbidity, as those only required data on the EQ-5D-5L descriptive system, age and gender, all of which was available for the whole of the final sample.

**P8, Paragraph 2. I'd agree that the level of missingness in EQ5D is low enough that a complete-case analysis here is ok.**

Great, thanks for confirming.

REVIEWER #2:

**We appreciate the opportunity to review the study entitled "The impact of the COVID-19 pandemic on health-related quality of life: a cross sectional survey of 13 high and low-middle income countries". We believe it addresses a relevant and pertinent issue. The objective of analysing the impact of the COVID-19 pandemic on HRQoL in different countries is fully justified.**

**The paper provides evidence on the impact of the pandemic on perceptions of health status and highlights the impact on mental health as a key element of this impact. These results are aligned with previously available evidence (Poudel et al Plos One 2021; Nguyen et al J Clin Med 2020,…), but the authors also adjust the effect studied for important personal and contextual factors, and offers an overview of different countries.**

We thank the Reviewer for appreciating the importance of our study and for their constructive suggestions, which have helped us to improve the quality of our manuscript.

**However, the study is not without limitations which need to be discussed.**

**Our main concerns are related to the so-called "recall bias" and to the utilization of the the measurement tool.**

**The "recall bias" appears to be significant and it depends on the event being recalled, time since the event, and patient clinical and demographic characteristics. HRQoL seems to be particularly sensitive to this bias. (Schmier et al Expert Rev. Pharmacoeconomics Outcomes Res. 2004). Patients tend to remember their baseline HRQoL as being better than it actually was (Litwin et al, J Clin Oncol 1999). Some of the references used by the authors have measured recall bias in short periods of time, two weeks (Lawson A et al, BMC Musculoskelet Disord 2020). Other studies, such as the one mentioned in the article by Haagsma J, et al (Health and Quality of Life Outcomes. 2019) found no differences over longer time periods, i.e. they did not report a significant recall bias at the group level, but this was due to random error, which did not guarantee the reliability of the data.**

**In this study, we consider this bias to be an important limitation, as the questions referred to a period of time that could be almost a year, and in the midst of a situation, the pandemic, that was having a profound social impact. "Recall bias" has been found so crucial in the measurement of HRQoL that adjustment measures have been proposed to "correct" the results, particularly using the EQ-5D (McPhail S et al, Health Qual Life Outcomes 2010).**

The Reviewer raises a very important limitation of our study, which we had already acknowledged in the original manuscript. However, we do recognise that a more in depth discussion is appropriate and, in fact, necessary.

Firstly, we have acknowledged this limitation up-front in the Abstract (page 2, lines 48-50):

*<<A limitation of the study is that subjects were asked to complete the pre-pandemic health questionnaire retrospectively, meaning responses may be subject to recall bias>>.*

Secondly, we have considerably expanded our original discussion of recall bias being a potential limitation within our study, drawing on the helpful articles that the Reviewer suggested, so to give a more nuanced view of the issue.

The revised discussion reads as follows on pages 14/15, lines 327-353, of the revised manuscript (please note that what we report below addresses also other subsequent points raised by the Reviewers, as we detail after each point is raised later in our response:

Pages 14/15, lines 327-353:

*<<Caution needs to be exerted when interpreting our findings. Findings may be biased by the fact that subjects were asked to complete the health questionnaire retrospectively and, therefore, responses may be subject to ‘recall bias’. Recall bias is a potentially complex phenomenon whereby, depending on context, people may over-weight salient events in the past or under-weight previous health problems with regards to their current health and demographic variables [27]. Current health may act as a lens or filter which affects how people recall the past [28]. The literature on recall accuracy using the EQ-5D is limited, and studies tend to be for specific populations and conditions [29-31]. Patients have also been found to recall their baseline HRQoL as being better than it actually was [32], which, in our study, would suggest the differences we observed within the first year of the COVID-19 pandemic were conservative. Furthermore, retrospective EQ-5D has been used previously in surveys for both COVID-19 [33, 34] and other diseases [35]. Compared with pre-pandemic norms in countries where EQ-5D-5L population norms are available, in Australia [36], Canada [37], China [38], Colombia [39], Spain [40], and the US [6], our retrospective HRQoL values are relatively low, even when the country-specific value sets were used (i.e. the US). If there is some downward bias in our baseline population average HRQoL estimates, all else equal, this may at least have the advantage of helping to mitigate any upward bias in our estimates of HRQoL fall. However, it also suggests that our sample may not be as representative as hoped, although HRQoL measures based on online responses have been found to be lower than face-to-face responses [6], and none of the aforementioned population norms are based on online responses. It is also worth noting that the EQ-5D-5L asks individuals to rate their current health status at the time of the survey [8], meaning responses are likely to be influenced by the context of the pandemic in each country at the time of the survey, conducted between 24 November 2020 and 17 December 2020. Given that this period coincided with a new wave of COVID-19 cases and deaths globally [41], respondents may have perceived their HRQoL as lower than they would have at other points during the pandemic, when new cases and deaths were lower>>.*

**Furthermore, the fact that the population norm expresses a better HRQoL status in a country than the selected sample from that country leads us to think about how good the representativeness of the sample was, but does not ensure that the bias does not overestimate the loss of HRQoL. Aditionally, if this situation is a constant in most of the countries studied, it would be worth noting (population norms for the EQ-5D exist in several of the countries studied, Australia, Colombia, China, UK, Canada, Spain...).**

The Reviewer is right in noting all of this. We have now edited the text, in the discussion, when talking about bias not overestimating the loss of HRQoL, as this may not be always the case. We have also compared our pre-pandemic EQ-5D index values with population norms in countries where EQ-5D-5L population norms are available, finding that our values are consistently lower than population norms, even when the same value sets were used (i.e. the US). We have made this clear in the discussion by contemplating the possibility that representativeness may play a role in our results, while also pointing to evidence that population norms based on online surveys have been found to be lower than those based on face-to-face surveys. This is all spelled out in the revised manuscript, on page 14, lines 338-347, and reported below:

<<*… Compared with pre-pandemic norms in countries where EQ-5D-5L population norms are available, in Australia [36], Canada [37], China [38], Colombia [39], Spain [40], and the US [6], our retrospective HRQoL values are relatively low, even when the country-specific value sets were used (i.e. the US). If there is some downward bias in our baseline population average HRQoL estimates, all else equal, this may at least have the advantage of helping to mitigate any upward bias in our estimates of HRQoL fall. However, it also suggests that our sample may not be as representative as hoped, although HRQoL measures based on online responses have been found to be lower than face-to-face responses [6], and none of the aforementioned population norms are based on online responses>>.*

**The second question is related to the measurement tool. The EQ-5D-5L is a valid and reliable instrument to measure HRQoL. The authors are experts in the use of this tool, but they do not point out in the manuscript that the EQ-5D assesses the current perceived health status. In the context of acute illness, HRQoL may deteriorate significantly, but only temporarily. During the weeks in which the study was conducted, a new peak incidence of infection was occurring worldwide (more than 4 million new cases in Europe alone, COVID-19 Weekly Epidemiological Update), which may have had some influence on the results expressed. But beyond this circumstance, the perception of HRQL may have been more similar to the baseline, further away from the incidence peaks, which has had an extraordinary social impact.
We believe that the impact of the pandemic on HRQoL would be more realistically measured in the nadir periods of the spread of the virus, and especially when the social conditions experienced (and reported by the media) were returning to normal.
This does not detract from the value of the manuscript, but its conclusions must be assessed in the context in which the study was conducted.**

We are extremely pleased to hear that, despite the unavoidable limitations, the Reviewer finds our manuscript of value. Indeed, setting and coordinating such a complex study across 13 diverse countries spanning six continents would have been very challenging in “normal times”, but it has been much more complicated during the COVID-19 pandemic unfolding. Whilst it would not have been straightforward to establish when the COVID-19 nadir periods were, we did not have full control on when the survey would have gone live in the different countries, because our work progress was very much hindered by the unexpected and unpredictable pace of the COVID-19 pandemic. The latter had an impact also on the capacity of our study team, not only on the answers of the prospective respondents to our surveys.

Nonetheless, we agree with the Reviewer that the issues they raised are important and should be spelled out in the revised manuscript. We have therefore updated the text in the methods section to make clear, on page 6, lines 141-143, that:

<<*Participants rated their* ***current*** *health at the time of the survey,* ***as for the standard EQ-5D instrument, but also*** *retrospectively* ***(under EuroQol Agreement 159150)****, thinking to the pre-pandemic period*>>.

We have also introduced specific text to the discussion section, highlighting the impact of contextual issues, as we agree that it is important the reader understands responses to the EQ-5D-5L are likely to be influenced by the context of the pandemic in each country at the time of the survey. This can be found on pages 14/15, lines 347-353, and reads as follows:

*<< It is also worth noting that the EQ-5D-5L asks individuals to rate their current health status at the time of the survey [8], meaning responses are likely to be influenced by the context of the pandemic in each country at the time of the survey, conducted between 24 November 2020 and 17 December 2020. Given that this period coincided with a new wave of COVID-19 cases and deaths globally [41], respondents may have perceived their HRQoL as lower than they would have at other points during the pandemic, when new cases and deaths were lower>>.*

**Other issues that should be reviewed are listed below:**

**- Please, explain how were estimated lost QALYs due to deaths. Are they taken from reference 22?**

The Reviewer is correct, we took the estimate of the QALYs loss due to COVID-19-related death from the recent literature, in particular from what was, in the original manuscript, reference 22 (Hernando et al.), and that has now become, in the revised manuscript, reference 13. This is because that study reported the highest and lowest estimates of QALYs lost per death within the current literature, which allowed us to determine the widest possible range of values. We have now added a paragraph in the Introduction section to give some context to such estimates – please see page 4, lines 93-100, which read as follows:

<< *QALYs derived from standard life table methods, adjusting for comorbidities and EQ-5D-derived HRQoL, have been recently used to assess the value of a COVID-19-related death. A recent study [12] suggested a COVID-19- related death to equal around 5 QALYs lost, on average, across the UK, US, Canada Norway, and Israel. A QALY loss ranging between 3.2 and 6.5 QALYs, depending on assumptions on Standardized Mortality ratio and impact of comorbidities on HRQoL, was instead indicated in a Spanish study [13], while a Dutch study estimated that QALYs lost because of COVID-19 mortality are on average 3.9 per death for men and 3.5 for women [14]*>>.

We have also better explained the relevant procedure in the Methods section, on page 9, lines 208-213:

*<<* *Country-level QALY loss due to COVID-19-related death was estimated by drawing on the recent literature expressing the value of a COVID-19 death in terms of QALYs lost [12-14]. In particular, we used the highest and lowest estimates of QALYs lost per death [13], so to determine the widest possible range of values, and multiplied it by each of the confirmed country-level COVID-19 deaths a year into the pandemic [1, 18].>>.*

**- We are confident that the authors could handle more appropriate methods of analysis when contextual variables are included in addition to individual variables. It would be interesting to know how much of the variability of the response is determined by natural groupings (countries). On the other hand, it would be useful to show some data about the goodness of fit of the models and why they were chosen.**

Our regression analyses were intentionally simple, as our aim was purely to explore associations, without attempting to establish any potential causality and/or potential moderators/mediators of the estimated relationships. Those type of analyses would have required different methods and would have answered different research questions, beyond the aims of this study.

We have now added a sentence to clarify this in the Discussion section, after discussing relevant results. This can be found on page 19, lines 460-463, and reads as follows:

*<<Further research is needed to understand the extent to which the observed associations may be causal, and to better elucidate the potential mechanisms underlying those relationships (e.g. mediators and moderators), which was beyond the aims of this study>>.*

Furthermore, under the advice of Reviewer #1 (Statistician), we have now dropped Model 4 to which this Reviewer is referring. Even in the original manuscript, in fact, we had not commented much on that model, only mentioning it in a sentence in which we noted that it confirmed the results of other models (M1/2 and 3, where applicable).

However, what this Reviewer notices is certainly true – by construction, the contextual variables in S8 Table varied predominantly by natural groupings (i.e. countries), as it is also shown by the way in which their odd ratios and statistical significance changed as we moved from Models 1 (unadjusted) to Models 2 (adjusted by age and country). We have now emphasised this in the Results section related to the macro-indicators, on page 11, lines 266-268:

*<< Results on these macro-indicators reflected the wider context in which each country was at the time of the survey >>.*

**- Wherever possible, country-specific value sets should be used to estimate country-specific utilities.**

We used a common value set in the main analysis to ensure comparability in between country analysis, which we have now explained in more detail in the methods (page 7, lines 153-155). Furthermore, EQ-5D-5L value sets are only available for a subset of the 13 countries included in our study.

However, we take the Reviewer’s point, and in the revised manuscript we have also generated EQ-5D-5L indices using the newly released India value set, to ensure that sensitivity analyses were undertaken using a variety of value sets, spanning high-income (US) and middle-income (India) countries (page 7, line 155-157). Results remained consistent when using the India value set, as they did when using the US value set (page 12, line 295-297).

The relevant clarifications in the Method section can be found on page 7, lines 153-158 and read as follows:

*<< EQ-5D-5L indices, measuring HRQoL, were generated using the UK [23] value set in the main analysis to enable comparability between countries, and the US [24] and India [25] value sets in the sensitivity analyses, incorporating in this way value sets from both high-income and middle-income countries >>.*

The relevant additions in the Results section can be found on pages 12, lines 296-297:

*<< Results remained consistent when using the US value set (S13, S14, and S15 Table) and India value set (S16, S17, and S18 Table). )>>.*

The new tables reporting the new results using the India value set are Tables S16, S17, and S18 and can be found in the Supplementary information.

**- Surprising results need to be discussed further. For example, why do the authors believe that higher levels government effectiveness were associated with reduced likelihood of reporting deterioration of health for males, but the opposite for females/others? Why might government effectiveness be related to worsening health?**

We have now discussed this result, in light of recent empirical evidence, in the Discussion section on pages 18/19, lines 448-463, which read as follows:

*<<. Interestingly, higher levels of government effectiveness were associated with reduced likelihood of reporting deterioration of health for males, but the opposite for females/others. It is possible that any positive relationship between government effectiveness and perceived health may be obscured by perception of effectiveness/trust in government. A recent report has found that in most OECD countries women have lower trust in the national government than men [47]. While a variety of causal mechanisms may drive this finding, women’s lower trust in government may derive from lower economic and educational opportunities or the existence of other structural gender inequalities in society. These may have been exacerbated during the COVID-19 pandemic, and may have contributed to trigger mental health’s deterioration, which in turn impacted on overall perceived health>>.*

**- Government responses at national level and government performance indices deserves a more detailed explanation (it seems appropriate to include it in the supplementary material). As pointed out by the authors, some indices, for example those related to government intervention (Government Response Index, Containment and Health Index ...) varied with the spread of the virus while the impact of government action and its relation to the population's perception is conditioned by the overall situation. So, the "lockdown" eased during the months in which the study was conducted, but it was more severe (and different between countries) in the previous months. This situation should be highlighted in the discussion.**

Further details on the COVID-19 government responses at national level and government performance indices can be found in the explanatory notes below Table S2, in the Supplementary information, which we have now made clearer in the methods section, on page 7, line 165-166):

*<< Indices definitions and further details can be found in the Supplementary information below S3 Table>>.*

We have also highlighted in the Discussion section, when talking about Government responsiveness results, the important points made by the Reviewer. They can be found on page 18, lines 437-444, and read as follows:

*<< It is worth remembering, however, that governments’ interventional measures varied with the spread of the virus. The way in which the government action was associated with the population’s perception of their health, instead, was more likely influenced both by personal and wider contextual circumstances at the time of the survey, as well as by the comparison of both with what they had previously experienced. For example, strictness of lockdown had generally eased at the time of the survey with respect to the very beginning of the pandemic, although differences among countries existed, which may have affected observed associations>>.*

**- Loss of income, along with previous illnesses, were strongly associated with worsening health status. Could it not be discussed how much of the worsening health status may be due to the economic impact of the pandemic (perhaps mediated by worsening mental health)?**

We have now discussed what suggested by the Reviewer on page 17/18, lines 421-428, which read as follows:

*<< This may suggest that it is the immediate and unexpected loss of income that is associated with perceived worse health most, especially for those with poor job security [9,11,44,45],* ***the likely driver of the association being a deterioration of mental health****. Those with chronic diseases were more likely to report deterioration in HRQoL, which is a consistent finding in COVID-19 studies.* ***A potential explanation of this observed association may include delays in obtaining the necessary health care and medicines for specific conditions, together with the anxiety that these difficulties may have generated****>>.*

As previously explained, we have also added a sentence, at the end of the discussion of the different observed associations, to highlight the need of further research to shed further ‘empirical’ light on the mechanisms underlying all of the observed associations, which was beyond the aims of this study (but that can be specifically addressed in subsequent studies). This can be found on page 19, lines 460-463:

*<<Further research is needed to understand the extent to which the observed associations may be causal, and to better elucidate the potential mechanisms underlying those relationships (e.g. mediators and moderators), which was beyond the aims of this study>>.*

**- The representativeness of the selected populations is rather questionable. It is not plausible that there are more highly educated people in Africa than in Europe or in North America. While the authors acknowledge this possible bias, they should discuss at length how it might affect the results.**

We have been transparent throughout the manuscript about the study limitation in terms of sample representativeness, but following this Reviewer’s suggestions, we have emphasised that limitation even more - please see also our responses to previous comments that mentioned this point, which brought us to point the reader to this limitation in relation to other parts of the discussion section.

In response to this repeated observation, we have now made it clearer in the Methods section that the samples for India and Uganda are not nationally representative, on page 5, line 117-120, which reads as follows:

*<< Except for India and Uganda, where samples were not nationally representative, including mainly urban settings, quota sampling was adopted to obtain representative samples in terms of age, education, gender, and geography in each country>>.*

Furthermore, we have also made this clearer in the Discussion section, so that the reader is aware that results are not generalisable and need to be considered with caution. This can be found on page 15, lines 355-363, and reads as follows:

*<<While samples are generally representative on key socio-demographic/geographical factors in the included high-income countries, the same cannot be claimed for low/middle-income countries, with India and Uganda, for example, being primarily sampled from urban populations. This means that the included samples from these countries are not nationally representative, with the implication that the associated results are representative of a more educated than average, self-selected, population, whose material circumstances and health perceptions may differ substantially from the overall population. Findings related to these countries are therefore not generalisable to the whole population and need to be interpreted with caution>>.*

**In conclusion, we believe that this is a valuable study, the results of which may be of interest.  However, it would benefit from some improvements in the method (proposed above) and a broader discussion of some of the associations found and the limitations intrinsic to the study design.**

We thank the Reviewer for the overall appreciation of our work and the thorough feedback and comments, which we hope we have address to their satisfaction.

REVIEWER #3:

**This is an interesting and informative paper. I have following comments on the paper:**

We thank the Reviewer for appreciating the importance of our study and for their constructive suggestions, which have helped us to improve the quality of our manuscript.

**1.      My first suggestion is- please cite all the references within sentences. For example, you have cited references either outside full-stops (.) or outside commas (,) which do not look right.**

All references have now been cited within sentences, i.e. before the punctuation.

**2.      In the Methods section, under 'Procedures', you have mentioned- "QALYs lost at population levels were estimated from the UK-valued EQ-5D-5L and external data on population sizes." Could you please give justifications for this as the value sets may differ greatly by study country? This may affect validity of your analysis results.**

We have provided justification in the methods section (page 7, lines 153-155) for using a single value set, as this approach enables comparisons to be made in the results across countries. Furthermore, EQ-5D-5L value sets are only available for a subset of the 13 countries included in our study. We used the UK value set because it was the one that was first made available to value the EQ-5D descriptive system and, therefore, the most used in the literature before other value sets were gradually developed, which may facilitate comparisons.

However, we understand the Reviewer’s concerns, and in the revised manuscript we have also generated EQ-5D-5L indices using the newly released India value set, to ensure that sensitivity analyses were undertaken using a variety of value sets, spanning high-income (US) and middle-income (India) countries (page 7, line 153-157). Results remained consistent when using the India value set, as they did when using the US value set (pages 12, lines 296-298).

The relevant clarifications in the Method section can be found on page 7, lines 153-157 and read as follows:

*<<EQ-5D-5L indices, measuring HRQoL, were generated using the UK [23] value set in the main analysis to enable comparability between countries, and the US [24] and India [25] value sets in the sensitivity analyses, incorporating in this way value sets from both high-income and middle-income countries>>.*

The relevant additions in the Results section can be found on pages 12, lines 296-298:

*<<Results remained consistent when using the US value set (S13, S14, and S15 Table) and India value set (S16, S17, and S18 Table). Higher HRQoL decrements occurred among the younger age groups (S19 Table)>>.*

The new tables reporting the results using the India value set are Tables S16, S17, and S18 and can be found in the Supplementary information.

**3.      In the Methods section, under 'Statistical Analyses', in the last paragraph you have mentioned- "The resulting country level QALY loss due to COVID-19 pandemic-related morbidity was then expressed as a ratio of country-level QALY loss due to COVID-19 premature mortality, estimated by multiplying confirmed country-level COVID-19 deaths by current estimates of QALYs lost per death.(22-24)". Method explained in this sentence is not very clear to readers. Please expand how did you calculate QALYs lost per death for every country, to make it clear to readers?**

**4.      In addition to above comment, in Table 3, you have used same lower and upper QALYs lost per death values for every country. How do you justify this as we know this may differ in every country?**

As Reviewer’s comments 3 and 4 are strongly interrelated, we respond to them together. We took the estimate of the QALYs loss due to COVID-19-related death from the recent literature, in particular from reference 13, Hernando et al. This is because that study reported the highest and lowest estimates of QALYs lost per death within the current literature, which allowed us to determine the widest possible range of values. QALYs loss due to COVID-19 deaths are in fact not available for all countries in our samples, but using the highest and lowest estimates available in the current literature, we enhance the chances that values for other countries fall within these conservative upper and lower bound. We have now added a paragraph in the Introduction section to give some context to such estimates – please see page 4, lines 93-100, which read as follows:

<< *QALYs derived from standard life table methods, adjusting for comorbidities and EQ-5D-derived HRQoL, have been recently used to assess the value of a COVID-19-related death. A recent study [12] suggested a COVID-19- related death to equal around 5 QALYs lost, on average, across the UK, US, Canada Norway, and Israel. A QALY loss ranging between 3.2 and 6.5 QALYs, depending on assumptions on Standardized Mortality ratio and impact of comorbidities on HRQoL, was instead indicated in a Spanish study [13], while a Dutch study estimated that QALYs lost because of COVID-19 mortality are on average 3.9 per death for men and 3.5 for women [14]*>>.

We have also better explained the relevant procedure in the Methods section, on page 9, lines 208-213:

*<<* *Country-level QALY loss due to COVID-19-related death was estimated by drawing on the recent literature expressing the value of a COVID-19 death in terms of QALYs lost [12-14]. In particular, we used the highest and lowest estimates of QALYs lost per death [13], so to determine the widest possible range of values, and multiplied it by each of the confirmed country-level COVID-19 deaths a year into the pandemic [1,18]>>.*

**5.      In the Results section (page 10), you have reported - "For all other health domains, younger groups remained most impacted, but deterioration in health was, on average, greater for males (Figure S11)." Could you please explain the reasons why deterioration in health was greater for males than females?**

In the revised manuscript, we have rephrased the sentence indicated by the Reviewer so to make it clearer. The change can be found on page 12, lines 278-281, and reads as follows:

*<<For all other health domains, worsening was reported in greater percentage by younger groups, but the percentage of males who reported deterioration in health was, on average, greater than the corresponding percentage reported by females (S11 Figure)>>.*

We have also added in the discussion section a paragraph that offers a ‘tentative’ explanation of this result. It can be found on pages 16/17, lines 398-412, and reads as follows:

*<<The fact that the percentage of males who reported deterioration in EQ-5D domains other than anxiety/depression was greater than the corresponding percentage of females/others is in line with previous evidence, as a multi-country study of EQ-5D population norms [42], when investigating responses to the EQ-5D domains by gender, found that usually gender related odd ratios were in favour of men in terms of reported problems, but there were some exception in the domains of mobility, self-care, and usual activities in some countries. In the context of the COVID-19 pandemic, these findings may be attributed to gender differences in the questionnaire interpretation. It may in fact be possible that males were interpreting COVID-19 related restrictions (i.e. lockdowns) as limiting their mobility and usual activities, whereas females/others responded the questions in terms of the extent to which their physical and mental health conditions impacted their physical ability to be mobile and conduct daily activities. However, in the absence of a follow-up question in the survey tailored to ascertain this potential mis-interpretation of the EQ-5D questionnaire and the potential gender-related bias in interpretation, this remains only a speculation>>.*

**6.      In the Results section, under "HRQoL changes" (page 11), you have mentioned- "Only China showed non-statistically significant changes". Why? Do you know any reasons why China showed non-statistically significant changes?**

We discuss the finding that only China showed non-statistically significant differences in HRQoL in the Discussion section. Our results were consistent with another similar study using the EQ-5D, and explained in terms of different cultural perceptions of health, overall health status, age, and gender structures, as well as pandemic stages. The relevant section, on page 16, lines 385-388, reads as:

*<< Only in China did perceived overall health not significantly change in our sample. A similar result was reported in another study using the EQ-5D [10], and attributed to different cultural perceptions of health, overall health status, age, and gender structures, as well as pandemic stages>>.*

**7.      In Table 1, could you please explain why female and other gender categorised in one group? Also, what types of gender come under 'other', any examples? Please define 'other' gender in the body of the paper too so that readers are clear about it.**

We thank the Reviewer for giving us the opportunity to clarify this important point. Out of the 15,536 respondents, only 34 (0.2%) reported their gender as ‘Other’, which was a category approved by the University of Oxford Ethics Committee to include the gender of all individuals who did not identified either as ‘Male’ or ‘Female’.

We believed it was very important to keep these respondents rather than exclude them, but low numbers and the associated lack of statistical power prohibited separate analyses for this sub-group of our sample. Exploration of data suggested that there was not a clear pattern and in fact sample sizes were too small to draw any meaningful conclusion. We have added some text to explain this and define the category ‘Other’, on page 8, line 184-188:

*<< Because of small numbers, respondents who reported their gender as ‘Other’ were combined with those who identified as ‘Female’. Exploration of data suggested that there was not a clear pattern and in fact sample sizes were too small to draw any meaningful conclusion. The category ‘Other’ included all individuals who did not identified either as ‘Male’ or ‘Female’ >>.*

**8.      In Table 1, average age of respondents from Africa reported as 29.11 yrs but other continent over 42 yrs. I doubt the sample taken from Africa may be representative of the study population (may be due to that reason, income loss from Africa is very high (88.73%) compared to other countries, as shown in Table 1). What is your opinion about the representativeness of sample from Africa? How do you convince the sample from Africa is representative of the study population?**

In our study, the only African country was Uganda, and the median age of the Uganda population is about 16.7 (<https://www.statista.com/statistics/447643/average-age-of-the-population-in-uganda/>), which may explain the lower average age compared to other countries. But beyond this fact, we have been transparent throughout the manuscript about the study limitation in terms of sample representativeness and, following on from this Reviewer’s comment, we have emphasised that limitation even more - and not only in relation to Uganda, but also in relation to India, in fact.

We have now made it clearer in the Methods section that the samples for India and Uganda are not nationally representative, on page 5, line 117-120, which reads as follows:

*<< Except for India and Uganda, where samples were not nationally representative, including mainly urban settings, quota sampling was adopted to obtain representative samples in terms of age, education, gender, and geography in each country>>.*

Furthermore, we have also made this clearer in the Discussion section, so that the reader is aware that results are not generalisable and need to be considered with caution. This can be found on page 15, lines 355-363, and reads as follows:

*<<While samples are generally representative on key socio-demographic/geographical factors in the included high-income countries, the same cannot be claimed for low/middle-income countries, with India and Uganda, for example, being primarily sampled from urban populations. This means that the included samples from these countries are not nationally representative, with the implication that the associated results are representative of a more educated than average, self-selected, population, whose material circumstances and health perceptions may differ substantially from the overall population. Findings related to these countries are therefore not generalisable to the whole population and need to be interpreted with caution>>.*

**9.      Again in Table 1, in co-morbidities categories, one category is- 'Allergies', which is too vague to understand by readers. Can you please give some examples in a bracket what are covered under 'Allergies' so that readers are clearer about this?**

In the original survey, respondents were asked ‘Do you have any of the following underlying health conditions (tick all that apply)?’ with one of the responses being ‘Allergies’. We therefore do not have any examples of what is covered by ‘Allergies’, as it was left open to the interpretation of the respondent. However, we have added a footnote to Table 1, which clarifies to the reader that:

*<< The category ‘Allergies’ refers to any kind of allergy, such as food allergy (e.g. lactose, eggs), plants and flowers (e.g. daisy), hay-fever etc >>.*
