# Supplementary material for: The COVID-19 pandemic and health-related quality of life across 13 high- and low-middle-income countries: A cross-sectional analysis
Source: PLoS Med. 2023 Apr 11;20(4):e1004146. doi: 10.1371/journal.pmed.1004146 (PMC10089360; doi:10.1371/journal.pmed.1004146)
Supplement: Attachment — Submitted filename: Response to editors and reviewers_R2.docx [file pmed.1004146.s034.docx]

**Manuscript PMEDICINE-D-22-01975R2: Responses to Editors’ and Reviewers’ comments**

**NOTE:** Editors’ and Reviewers’ comments are shown in **bold**, with our responses shown below each. We also report in our responses relevant edited extracts from the revised manuscript in *Italics*.

------------------------------------------------------------
**Requests from Editors:**

**Thank you for including an Author Summary - this should immediately follow the Abstract in your revised manuscript.**

Thank you for noting this - we have now placed the Author Summary immediately after the Abstract on pages 3 and 4, lines 57-91, of the revised manuscript.

**Please combine bullets one and two in your Author Summary into a single bullet (“The health burden of the COVID-19 pandemic has been closely tracked in terms of confirmed cases and deaths due to the virus, but data on the relationship between the COVID-19 pandemic and health-related quality of life of the general population globally is still quite limited”) to ensure the Author Summary matches our formatting guidelines.**

Thank you for your advice, which we have now implemented by combining original bullets points one and two into a unique bullet point in the revised Author Summary on page 3, lines 59-62 of the revised manuscript.

**Please change ‘due to’ to ‘associated with’ in bullet four of you current Author Summary to reflect the observational nature of the study.**

We are unsure about which “due to” the Editors refer. Bullet point four in the original Author Summary read:

*<< It is important to understand similarities and differences* ***in the association between the COVID-19 pandemic and health-related quality of life*** *in different countries around the world, because this information can help policy-makers to find the most appropriate interventions that work in diverse settings, health conditions, and populations, to combat the ongoing COVID-19 pandemic and potential future pandemics>>*

so, there was no use of the wording “due to”.

We have also re-checked all the parts of the Author Summary, and we can confirm that we never used the wording “due to” in relation to our observational study results. The only instances in which we used “due to” were in relation to COVID-19 confirmed cases and deaths reported in sources external to our observational study. We would however be happy to further modify the Author summary if the Editors could indicate where specifically the changes are necessary.

The revised version of the Author Summary, as reported on pages 3 and 4, lines 57-91, of the revised manuscript, now reads as follows:

<< ***Author Summary***

***Why was this study done?***

- *The health burden of the COVID-19 pandemic has been closely tracked in terms of confirmed cases and deaths* ***due to*** *the virus, but data on the relationship between the COVID-19 pandemic and health-related quality of life of the general population globally is still quite limited.*
- *Existing studies have mainly focussed on single countries and used relatively small convenience samples, making cross-country comparison difficult.*
- *It is important to understand similarities and differences in the association between the COVID-19 pandemic and health-related quality of life in different countries around the world, because this information can help policy-makers to find the most appropriate interventions that work in diverse settings, health conditions, and populations, to combat the ongoing COVID-19 pandemic and potential future pandemics.*

***What did the researchers do and find?***

- *We asked 15,536 participants from a diverse group of 13 countries (approx. 1,200 per country) to answer an online survey in which we asked questions about socio-demographic characteristics, health, and perceived health-related quality of life prior to and during the pandemic.*
- *We found that the COVID-19 pandemic was associated with significantly worse health-related quality of life for more than one third of respondents, with anxiety/depression being the aspect of health that worsened the most, especially for younger people (<35 years) and females.*
- *Overall, our results suggest that there was an 8% reduction in perceived health-related quality of life, and the health burden, measured in quality-adjusted life years, associated with morbidity was considerably higher than that* ***due to*** *premature, COVID-19-related, mortality.*

***What do these findings mean?***

- *The health burden of the COVID-19 pandemic may be substantially underestimated if we consider mortality alone.*
- *Health-related quality of life measures are important to fully capture the considerable health burden of the COVID-19 pandemic and its associated containment policies.*
- *Our results provide benchmark evidence for countries at different stages in the pandemic and can help inform public health measures and economic policies in the event of other health shocks in the future >>.*

**Thank you for your explanation as to why those identifying as neither male nor female were grouped with females for the analysis. I would suggest updating the additional sentence at line 184 to ‘Because of small numbers, the respondents who reported their gender as ‘Other’ were arbitrarily combined with those who identified as ‘Female’ for the purposes of the analysis’.**

Thank you for your suggestion – we have now edited the relevant sentence accordingly. This can be viewed on page 9, lines 220-222, of the revised manuscript.

**Related to the above, I’d recommend adding footnotes to Table 1 and Figure 2 briefly explaining the grouping of ‘Female & Other’.**

We agree that this is important and we have now added footnotes to Table 1, on page 29 of revised manuscript, and Figure 2, on page 31 of the revised manuscripts. Both explanatory footnotes read as follows:

*<<Because of small numbers, the respondents who reported their gender as ‘Other’ were arbitrarily combined with those who identified as ‘Female’ for the purposes of the analysis>>.*

**Line 96 is missing a comma between Canada and Norway.**

We have now added a comma between Canada and Norway on page 6, line 132, of the revised manuscript.

------------------------------------------------------------
**Comments from Reviewers:**

**Reviewer #1:** **Thanks for the revised manuscript and responses to my review. Apologies for missing for some of the details in my first revision, the manuscript explains or link to explanations about the study sample and pre-registered protocol.

One of the other reviewers also noted the interpretation of change in HRQoL based on recall. I think that the limitations of this are fully explained in the manuscript now. While having a time machine to gather this information pre-COVID would be nice, recall is the next best thing and the authors have been clear about what they have collected and the limitations in interpretation.

The part looking at association between worsened health and individual/country characteristic and experience of COVID looks good to me now, I found the new version easy to follow and understand without the model that had the additional backward selection. With a relatively large sample size using p<0.10 for screening should be ok here.

Happy to recommend that the paper is accepted.**

**Reviewer #2:** **Thank you for allowing us to evaluate the revised version of this interesting manuscript.

We consider that almost all the issues raised have been addressed in a fully satisfactory way. We remain of the opinion that other methods of analysis could provide valuable additional information on the variability of response across countries, but the authors have chosen otherwise.

We are also aware of the limitations of the study in translating its conclusions into decision-making or assessing health situations such as those studied. But the authors have used the available tools in an absolutely accurate and honest way. While the manuscript was certainly valuable in its original presentation, we believe that the authors have improved many important aspects in this review. They have explained, throughout the different sections of the manuscript, all these limitations and their possible implications, so we only have to thank them for their work.**

**Reviewer #3:** **The authors have addressed all of my comments. Therefore, I am happy the editorial team take further steps for publication. Thank you.**

We would like to thank again each of the three Reviewers for taking the time to review our manuscript and for recommending its publication. Their insightful comments and suggestions on the previous version of our manuscript have helped us to improve the quality of our article.
